# Centromere-Like Regions in the Budding Yeast Genome

**DOI:** 10.1371/journal.pgen.1003209

**Published:** 2013-01-17

**Authors:** Philippe Lefrançois, Raymond K. Auerbach, Christopher M. Yellman, G. Shirleen Roeder, Michael Snyder

**Affiliations:** 1 Department of Molecular, Cellular, and Developmental Biology, Yale University, New Haven, Connecticut, United States of America; 2 Program in Computational Biology and Bioinformatics, Yale University, New Haven, Connecticut, United States of America; 3 Howard Hughes Medical Institute, Yale University, New Haven, Connecticut, United States of America; 4 Department of Genetics, Yale University, New Haven, Connecticut, United States of America; 5 Department of Genetics, Stanford University School of Medicine, Stanford, California, United States of America; University of Minnesota, United States of America

## Abstract

Accurate chromosome segregation requires centromeres (*CEN*s), the DNA sequences where kinetochores form, to attach chromosomes to microtubules. In contrast to most eukaryotes, which have broad centromeres, *Saccharomyces cerevisiae* possesses sequence-defined point *CEN*s. Chromatin immunoprecipitation followed by sequencing (ChIP–Seq) reveals colocalization of four kinetochore proteins at novel, discrete, non-centromeric regions, especially when levels of the centromeric histone H3 variant, Cse4 (a.k.a. CENP-A or CenH3), are elevated. These regions of overlapping protein binding enhance the segregation of plasmids and chromosomes and have thus been termed Centromere-Like Regions (*CLR*s). *CLR*s form in close proximity to *S. cerevisiae CEN*s and share characteristics typical of both point and regional *CEN*s. *CLR* sequences are conserved among related budding yeasts. Many genomic features characteristic of *CLR*s are also associated with these conserved homologous sequences from closely related budding yeasts. These studies provide general and important insights into the origin and evolution of centromeres.

## Author Summary

Centromeres (*CEN*s) are chromosomal regions essential for proper chromosome segregation through their ability to establish evolutionarily conserved protein complexes called kinetochores. During mitosis, kinetochores attach to microtubules emanating from spindle poles, thus providing the mechanism for chromosome segregation. Eukaryotes have different types of *CEN*s. Most eukaryotes have large multimeric centromeres lacking DNA sequence specificity. In contrast, the budding yeast, *S. cerevisiae*, has short punctate centromeres, comprised of specific DNA sequences. Combining chromatin immunoprecipitation and deep sequencing, we identified regions of the yeast genome that are bound by key kinetochore components; we refer to these regions as Centromere-Like Regions (*CLR*s). We found that *CLR*s can promote segregation on episomal plasmids and native chromosomes. Most *CLR*s are found in intergenic regions, close to native *CEN*s. *CLR*s resemble point *CEN*s by their short size and regional centromeres by their lack of determining DNA sequences. *CLR* sequences are conserved among related budding yeasts. Our findings indicate that, similar to other fungi and eukaryotes, *S. cerevisiae* possesses the ability to form sequence-independent centromeric structures. Establishment of centromeric elements outside regular *CEN*s, or neocentromerization, can lead to chromosome missegregation and is a hallmark of cancer cells. *CLR* formation in budding yeast provides a simple model of neocentromerization.

## Introduction

The kinetochore is a conserved proteinaceous structure that assembles on centromeric DNA and is responsible for connecting chromosomes to the spindle, thus ensuring accurate chromosome segregation. The length of centromeric DNA differs among eukaryotes, from less than one kilobase pair (kb) to several megabase pairs (Mb) [1]. This variation is most striking in fungi: whereas most fungi have large, regional centromeres spanning several kbs, the *Saccharomyces* lineage has small, punctate *CEN*s encompassing only 125 base pairs (bp) [2]. A hallmark of centromeric chromatin is the presence of the histone H3 variant CENP-A, or CenH3 [1], known as Cse4 in *Saccharomyces cerevisiae* [3]. Overproduction of human CENP-A promotes its incorporation onto non-centromeric loci and has been linked to colorectal cancer and aneuploidy [4]. In *S. cerevisiae*, Cse4 is commonly found outside centromeres using chromatin immunoprecipitation coupled to high-throughput sequencing (ChIP-Seq) [5]. Whereas overproduction of Cse4 does not appear to be severely deleterious or lead to a decrease in cell viability in yeast [6], it can become lethal in the absence of its specific E3 ubiquitin ligase Psh1 due to massive and stable Cse4 euchromatin incorporation [7], [8].

If Cse4 accumulation at non-centromeric sites is functional, i.e. imparts centromere-like activity, then additional kinetochore proteins should also be present. To investigate this possibility, we generated genome-wide binding profiles using ChIP-Seq to characterize four epitope-tagged kinetochore proteins, comparing a wild-type strain with normal levels of Cse4 (WT) to a strain overproducing Cse4 (Cse4 OP). Our ChIP-Seq data indicate recruitment of all tested kinetochore proteins to discrete sites outside *CEN*s, termed Centromere-Like Regions (*CLR*s). We showed that cloned *CLR*s can help the segregation of a *CEN*-less episomal plasmid and that endogenous *CLR*s can promote accurate segregation of a chromosome bearing an inactivated centromere. We found that most *CLR*s are found in larger than average intergenic regions and lie in close proximity to *S. cerevisiae* centromeres. Other genomic features associated with *CLR*s include a weak association to autonomously-replicating sequences (*ARS*; yeast origins of DNA replication) and an increased level of “AT” nucleotides over a short stretch of DNA. We observed sequence conservation of *CLR*s with members of the *Saccharomyces sensu stricto* and other budding yeasts carrying point *CEN*s, but not with other yeasts and fungi bearing larger, regional *CEN*s. Our results have implications for the origin and evolution of centromeres since *CLR*s might constitute evolutionary remnants from regional *CEN*s.

## Results/Discussion

### Identification of *CLR*s using ChIP–Seq

ChIP-Seq data were generated for four epitope-tagged kinetochore proteins: Cse4 (CenH3), the outer kinetochore protein Ndc80 (Hec1), and the inner kinetochore components Mif2 (CENP-C) and Ndc10 (Cbf2). We compared a wild-type strain with normal levels of Cse4 (WT) to a strain overproducing Cse4 from the Gal1–10 promoter (Cse4 OP), with at least a 3-fold increase in Cse4 protein levels in Cse4 OP as measured by Western blots (Figure S1; Cse4 with 3HA epitope as an internal tag). All proteins were tagged at their native locus and were the only copies present in the haploid cell. At least two biological replicates were examined per tagged strain, and these were compared to a matched control representing an immunoprecipitate from an untagged strain [9]. Regions of significant binding were identified with the PeakSeq algorithm using a stringent q-value threshold of 10^−5^ [10], [11] and further filtered to remove regions of poor enrichment.

**Figure 1:**
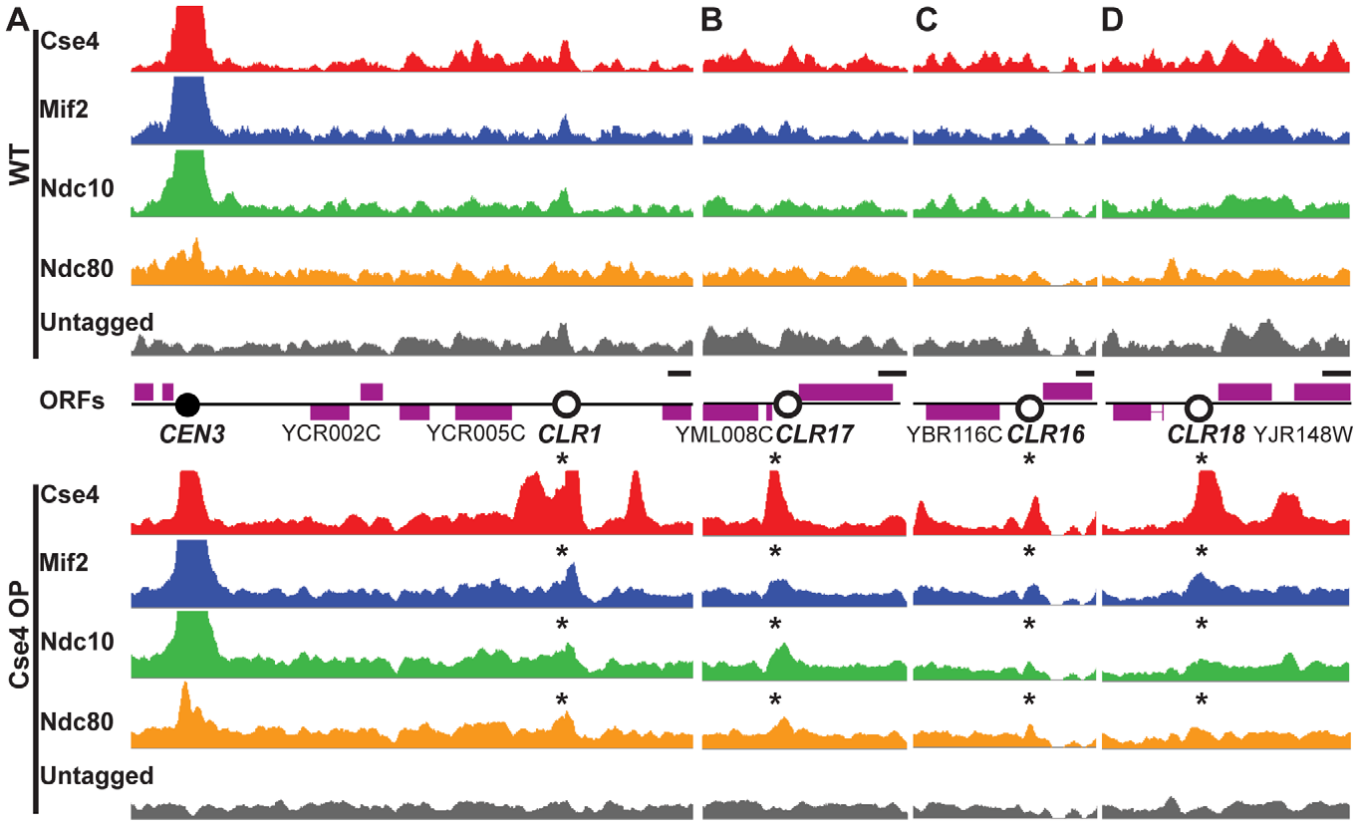
Formation of Centromere-Like Regions revealed by ChIP–Seq. Cse4 (red), Mif2 (blue), Ndc10 (green) and Ndc80 (orange) bind to the same discrete regions outside centromeres in Cse4 OP (bottom panels), but not in WT (top). (A–B) Highest-confidence sites are *CEN*-proximal; examples include *CLR*s 9 kb away from *CEN3* (A), and 15 kb from *CEN13* (B). Asterisks above signal tracks denote location of *CLR*s. (C–D) A few sites are far from *CEN*s; examples include *CLR*s 239 kb away from *CEN2* (C) and 267 kb from *CEN10* (D). Signal tracks are scaled relative to the number of uniquely-mapping reads. Control samples (immunoprecipitates from untagged strains) are shown in grey. Open reading frames (ORFs) are depicted by purple boxes. Horizontal scale bars represent 0.5 kb.

**Figure 2:**
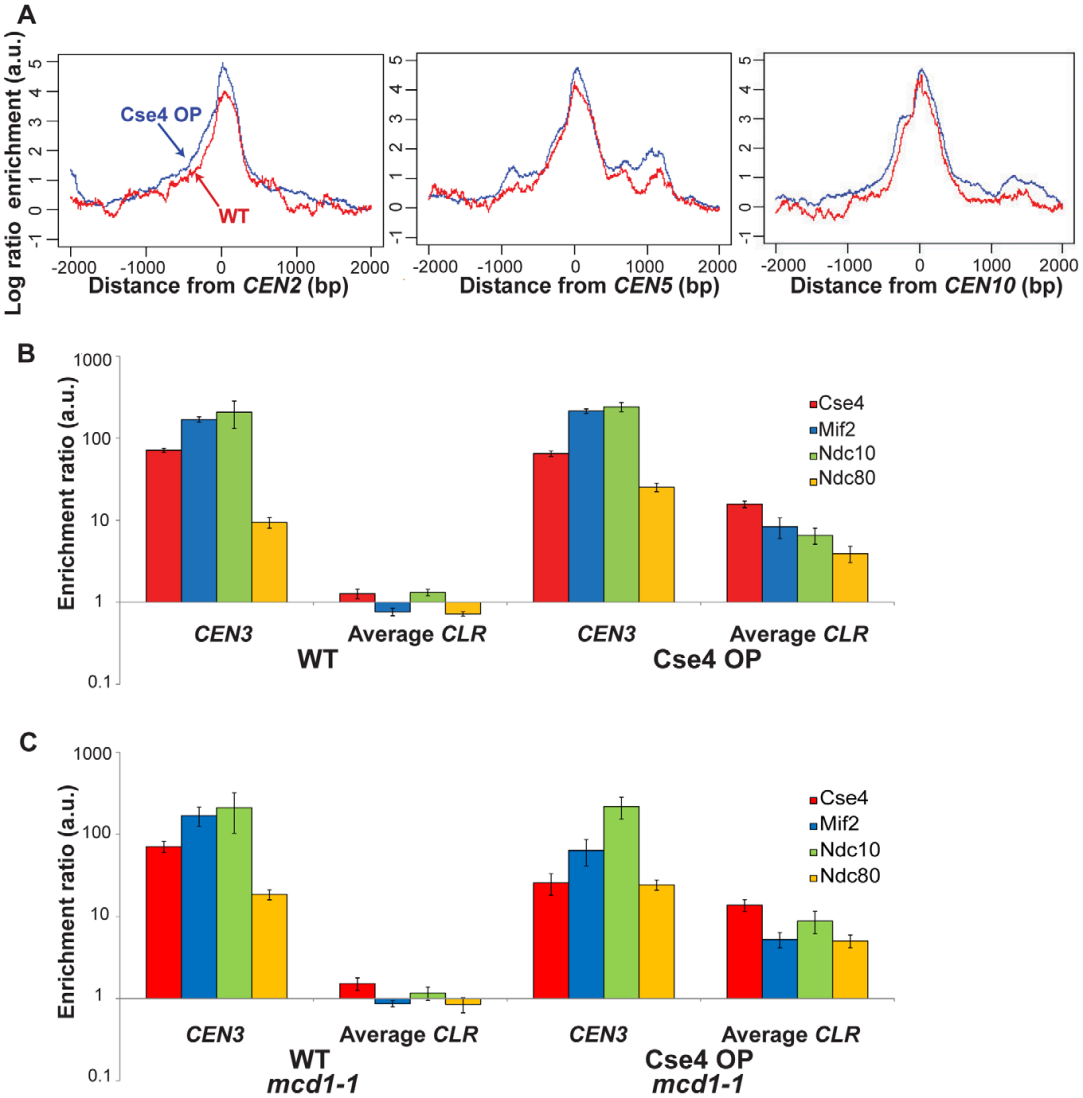
Quantitation of protein binding in Cse4 OP strains. (A) Kinetochore proteins show a broader distribution at centromeres when Cse4 is overproduced. Shown is ChIP-Seq signal for kinetochore proteins in Cse4 OP (blue) compared to WT (red) at *CEN2* (left), *CEN5* (middle) and *CEN10* (right). Aggregated signal plots depict the log ratio of read enrichment for four kinetochore components, centered at the *CEN*, on log 2 scales. (B) ChIP-qPCR confirms the presence of kinetochore proteins at *CLR*s in Cse4 OP, not in WT. Individual protein enrichments for 6 *CLR*s were averaged and compared to *CEN3* binding levels. Normalized enrichment ratios (means in arbitrary units (a.u.)+/−SEM) were plotted on a log 10 scale. A normalized enrichment of 1 indicates no enrichment over a negative control region not enriched for kinetochore proteins. (C) ChIP-qPCR in a cohesin-deficient *mcd1-1* background highlights *CLR* formation in Cse4 OP despite the abrogation of the pericentric intramolecular C loop. Individual protein enrichments for 6 *CLR*s were averaged and compared to *CEN3* binding levels. Normalized enrichment ratios (means in arbitrary units (a.u.)+/−SEM) were plotted on a log 10 scale.

Consistent with the presence of sequence-defined point centromeres in *S. cerevisiae* [2], Cse4, Mif2, Ndc10 and Ndc80 bind very strongly to *CEN*s in WT and Cse4 OP strains (Figure 1A and Figure S2). Overproduction of Cse4 generates a broader ChIP-Seq signal for kinetochore proteins at some centromeres, which is particularly apparent in aggregated signal plots around *CEN2*, *CEN5* and *CEN10* (Figure 2A and Figure S2; P = 0.03; paired t-test) and is consistent with ChIP-qPCR data from *S. cerevisiae* [12]. A similar pattern has been observed in the pathogenic budding yeast *Candida albicans*, where Cse4 overproduction is associated with the presence of extra kinetochore proteins and microtubules at *CEN*s [13].

In WT, only centromeric regions exhibit significant overlapping binding among all four tested kinetochore components (Figure 1, top). However, in Cse4 OP, several non-centromeric locations display overlapping binding, albeit to a lesser extent than native *CEN*s (Figure 1 bottom). We termed these 23 non-centromeric loci Centromere-Like Regions, or *CLR*s (Table 1). There is a strong bias towards formation of *CLRs* in close proximity to centromeres; about half lie within 25 kb of a *CEN* (P<10^−5^; randomization test), especially among those displaying high levels of protein binding (Figure 1A–1B). Most chromosomes have at least one centromere-proximal *CLR*. A few *CLR*s are located far from the actual *CEN* (>100 kb distal), and, compared to *CEN*-proximal *CLR*s, these centromere-distal *CLR*s are generally associated with reduced, yet significant, occupancy of the outer kinetochore protein Ndc80 (Figure 1C–1D).

**Table 1:** Chromosomal coordinates of Centromere-Like Regions (*CLR*s) and of sites that did not pass statistical filters (low-confidence, negative control regions (*LCNCR*s)).

Site	Chromosome	Start	End
***CLR1***	3	123200	124000
***CLR2***	14	631600	632200
***CLR3***	6	221500	222200
***CLR4***	13	306300	307000
***CLR5***	9	356900	357600
***CLR6***	13	271700	272200
***CLR7***	4	1013700	1014300
***CLR8***	8	103900	104500
***CLR9***	5	140400	141000
***CLR10***	1	141500	142000
***CLR11***	8	125800	126400
***CLR12***	11	230600	231200
***CLR13***	4	465000	465800
***CLR14***	15	978300	979500
***CLR15***	11	518300	518900
***CLR16***	2	477000	477600
***CLR17***	13	253100	253800
***CLR18***	10	703200	704000
***CLR19***	7	483200	484000
***CLR20***	13	751300	751800
***CLR21***	5	305800	306400
***CLR22***	6	4600	5100
***CLR23***	11	666000	666600
***LCNCR1***	7	1000900	1001700
***LCNCR2***	6	253500	255300
***LCNCR3***	3	137600	139100
***LCNCR4***	15	159400	160400
***LCNCR5***	11	326000	327300
***LCNCR6***	7	883300	884000
***LCNCR7***	8	98300	99300
***LCNCR8***	16	550400	551900
***LCNCR9***	3	91300	92400
***LCNCR10***	7	507900	508600
***LCNCR11***	16	645600	646500
***LCNCR12***	11	163500	164600
***LCNCR13***	13	476000	477600
***LCNCR14***	5	291900	293000
***LCNCR15***	12	459700	460800
***LCNCR16***	12	232000	234500
***LCNCR17***	4	555700	556600
***LCNCR18***	15	779900	781200
***LCNCR19***	4	312700	314200
***LCNCR20***	12	370500	371000
***LCNCR21***	2	274100	281100
***LCNCR22***	13	220000	221200
***LCNCR23***	13	861100	861800
***LCNCR24***	8	451300	452700
***LCNCR25***	12	290100	292500
***LCNCR26***	11	345400	346500
***LCNCR27***	1	71700	73300
***LCNCR28***	2	373600	375800
***LCNCR29***	2	612500	614000
***LCNCR30***	12	369600	370400
***LCNCR31***	14	559200	559900
***LCNCR32***	4	600700	602200
***LCNCR33***	12	1064700	1065300
***LCNCR34***	15	29400	33900
***LCNCR35***	5	138400	138900
***LCNCR36***	4	461600	462600
***LCNCR37***	5	139000	139900
***LCNCR38***	12	837500	839300

Protein binding was validated at six different *CLR*s and at *CEN3* by ChIP-qPCR. In WT, no individual *CLR* showed significant binding (normalized enrichment ratio >2) for all four proteins (Figure 2B and Figure S3A). However, in Cse4 OP strains, binding of four kinetochore components was significant at each of six *CLR*s tested (Figure 2B and Figure S3B). Protein occupancy at *CLR*s is about an order of magnitude less than levels seen at *CEN3* (Figure 2B), confirming that *bona fide CEN*s remain the primary sites where kinetochore proteins reside in budding yeast with elevated Cse4 abundance.

Pericentric chromatin is arranged in an intramolecular C loop that extends >25 kb but <50 kb around *CEN*s [14], generating the mitotic centromere spring that balances tension at the metaphase plate from the spindle microtubules [15], [16]. This loop arrangement requires cohesin, as loss of cohesion using the *mcd1-1* allele at the restrictive temperature abrogates the pericentric loop [14]. In this C loop configuration, centromeres and sequences from proximal regions might be in close spatial proximity; a possible consequence might be that kinetochore proteins are deposited onto *CEN*-proximal *CLR*s due to crosslinking and spatial proximity. To rule out this possibility, and also to determine the dependence of *CLR* formation on the pericentric loop, we repeated ChIP-qPCR analyses but in a cohesin-deficient *mcd1-1* background. In strains with normal Cse4 levels (WT) in *mcd1-1*, results remained unchanged; no individual *CLR* showed significant binding for all four proteins (Figure 2C and Figure S4A). In strains with elevated Cse4 levels (Cse4 OP) in *mcd1-1*, binding of four kinetochore components was significant at each of six *CLR*s tested (*CEN*-proximal and *CEN*-distal *CLR*s) and did not differ greatly from strains with functional Mcd1 (Figure 2C and Figure S4B). These results suggest that formation of *CEN*-proximal *CLR*s is not a biological artefact from simply crosslinking higher-order interactions, and that an intact cohesin-dependent pericentric loop was dispensable for *CLR* formation or, at the very least, for maintenance of kinetochore components at *CLR*s.

### *CLR*s exhibit centromeric activity on plasmids and chromosomes

**Figure 3:**
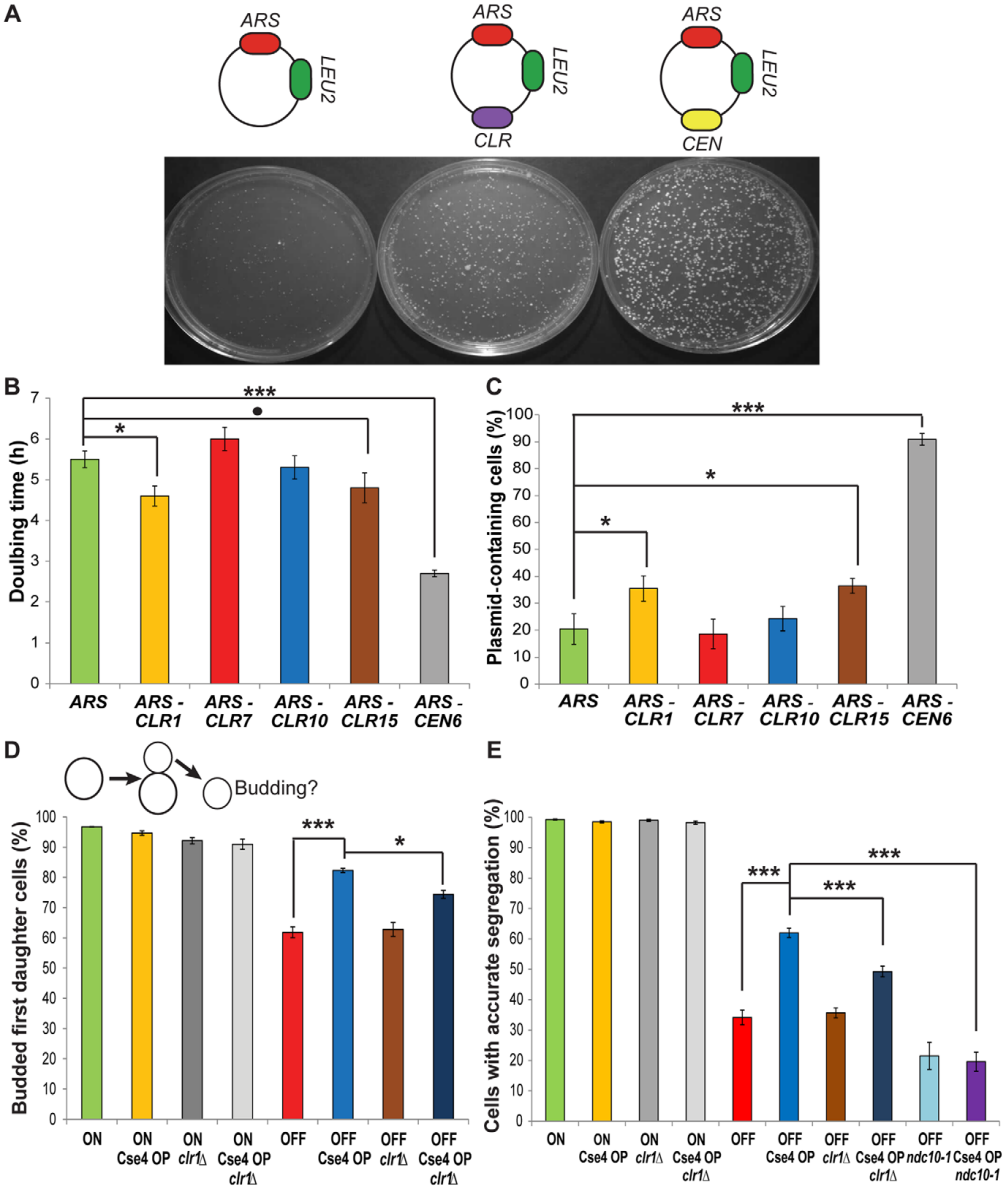
*CLR*s confer centromere function on plasmids and chromosomes. (A) Top: Representation of *ARS*, *CLR* and *CEN* plasmids. Bottom: Transformation plates from strains carrying different plasmids. (B) Doubling times in selective medium of strains carrying different plasmids (Means+/−SEM). (C) Fraction of plasmid-containing cells after growth in non-selective media for strains bearing various plasmids (Means+/−SEM). P-values were computed using MCMC simulations (* p<0.05, ** p<0.01, *** p<0.001,. p = 0.07) (B–C). (D) Top: Depiction of pedigree assay. Unbudded mother cells contain a conditional *CEN3* (ON raffinose; OFF galactose) or not (ON raffinose or galactose). Bottom: Fraction of budded daughter cells 12 h after transfer of mother cells to plates containing galactose (Means+/−SEM). ON and OFF indicate the presence of conditional *CEN3*. For the four left-most bars, similar values were obtained for strains with conditional *CEN3* grown in raffinose (ON). A time course is shown in Figure S4. (E) Fraction of cells that segregated properly their GFP-labelled chromosome 3, as visualized by the presence of a GFP dot in each cell (Means+/−SEM). P-values have been calculated using Fisher's Exact Test (* p<0.05, ** p<0.01, *** p<0.001) (D–E).

To determine whether *CLR*s act like centromeres, four different *CLR* sequences were tested in plasmid and chromosome segregation assays [17]. First, we asked if a *CLR* can function as a centromere on a plasmid containing an *ARS*. An *ARS*-only plasmid can replicate, but it is unstable and lost at high frequency [18], [19]. Addition of a *CEN* to an *ARS* plasmid renders it stable and efficiently transmitted to daughter cells [2]. *CLR* sequences were cloned into an *ARS* plasmid, and *ARS-CLR* plasmids (*CLR* plasmids) were compared to *ARS*-only (*ARS* plasmid) and *ARS-CEN* plasmids (*CEN* plasmid) (Figure 3A). After transformation of a Cse4 OP strain, two of the four *CLR* sequences tested (*CLR1* and *CLR15*) produced colonies of intermediate size; these were larger than those obtained from *ARS* plasmids, but smaller than those from *CEN* plasmids (Figure 3A). Other *CLR* plasmids (*CLR7* and *CLR10*) behaved like *ARS* plasmids. Consistent with the requirement for Cse4 recruitment to extrachromosomal plasmids for their segregation [20]–[23], the two apparently functional *CLR*s, *CLR1* and *CLR15*, had higher enrichment values for Cse4 than *CLR7* and *CLR10* (mean PeakSeq ratios 5.05+/−0.78 vs. 2.41+/−0.17, similar trends with ChIP-qPCR). As another test of segregation proficiency, doubling times in selective medium (SC Raffinose/Galactose – LEU) were measured. *CLR1* and *CLR15* decreased doubling time compared to an *ARS* plasmid, but to a lesser extent than *CEN* plasmids (Figure 3B; MCMC simulation). To ask whether *CLR* plasmids are more stably maintained than *ARS* plasmids, we measured the fraction of cells that retained the plasmid after growth in non-selective medium (YPAU+Raffinose/Galactose) for ∼4 generations [17]. *CLR* plasmids were maintained in a significantly greater fraction of cells (35% for *CLR1*; 36% for *CLR15*) than *ARS* plasmids (20%) (Figure 3C; P = 0.036 for *CLR1*; P = 0.018 for *CLR15*; MCMC simulation). The *CEN* plasmid was maintained in 91% of cells (Figure 3C). Differences in colony sizes were observed upon plating on selective medium, similar to Figure 3A (Figure S5). To ensure that these observed differences in *ARS* and *CLR* plasmid stability did not result from a size-dependent increase in plasmid stability [24] when comparing *ARS-CLR* and *ARS*-only plasmids due to the additional insert, we repeated plasmid segregation assays (doubling times and plasmid retention) with *ARS* plasmids bearing random inserts of similar sizes to *CLR* inserts, respectively 1 kb for *ARS-R1* and 0.8 kb for *ARS-R2* (Figure S6). Statistical significance for *ARS-CLR* plasmids was re-assessed then in comparison to *ARS-R1* or *ARS-R2* and found to follow similar trends than those obtained with *ARS*-only plasmid as a control (Figure 3B–3C and Figure S6). Taken together, these results indicate that *CLR* sequences can enhance plasmid segregation.

Second, we asked if a *CLR* can function in its natural context, on a chromosome, to promote proper segregation. Galactose-driven transcription towards a native *CEN* inactivates the kinetochore, thus creating a conditional centromere that can be switched off when cells are grown in galactose [25]. Two chromosome segregation assays were used to assess the stability of chromosome 3, carrying a conditional *CEN3* and *CLR1*, the only naturally-occurring *CLR* on chromosome 3. First, segregation was monitored by pedigree analysis; bud emergence in a daughter cell was assayed after *CEN* inactivation in an unbudded mother cell (Figure 3D) [19]. Budding of a daughter cell indicates accurate segregation of the *CEN*-inactivated chromosome 3 in the previous mitosis [26]. When *CEN3* is active, 95% of daughter cells are budded, in WT and Cse4 OP strains (Figure 3D and Figure S7). In contrast, when *CEN3* is inactivated, significantly more daughters of Cse4 OP cells bud compared to WT (82% vs. 62%; P<10^−5^; Fisher's Exact Test (FET)). In a second assay, we followed segregation of a GFP-labeled chromosome 3 after a single nuclear division [17]. Normal equational chromosome segregation results in a single GFP dot in both cells, whereas improper segregation results in two GFP dots in the same cell. Accurate chromosome segregation dominates in both genotypes when *CEN3* is active (Figure 3E). Cse4 OP partially rescues the missegregation of a *CEN3*-inactivated chromosome (Figure 3E; P<10^−10^; FET). This improvement in faithful chromosome segregation is weaker than that provided by a natural centromere or by a physically-tethered synthetic kinetochore [17]. Our results indicate that Cse4 OP enhances proper segregation of a chromosome with an inactive *CEN*. In *C. albicans*, Cse4 overproduction improves segregation in mutants defective in kinetochore proteins Dam1 and Dad2 [13]. While highly unlikely given the level of rescue observed, there is still a possibility that complete *CEN3* inactivation might be hindered at a higher degree in Cse4 OP due to excess Cse4 molecules *per se*.

When *CEN3* is inactive, Cse4 OP significantly improves the segregation of chromosome 3. To test whether this improvement is due to *CLR* activity, we deleted *CLR1* by gene replacement and then monitored chromosome segregation by pedigree analysis and GFP imaging. In both assays, deletion of *CLR1* decreased the Cse4 overproduction-dependent rescue of chromosome segregation, by 46% in the case of the budding assay and by 39% for the GFP dots assay (Figure 3D–3E; P = 0.03 and P<10^−5^, respectively; FET). To ensure that the observed decreased was caused by the loss of *CLR1* and not by impacted kinetochore assembly at *CEN3* arising from the experimental manipulation (gene replacement at *CLR1*), we verified binding of Cse4, Mif2, Ndc10 and Ndc80 at *CEN3* in *clr1* strains for WT and Cse4 OP. We found that binding levels of kinetochore components at *CEN3* in *clr1* strains were similar to those in *CLR1+* strains, confirming that *CEN3* integrity is intact in *clr1* strains (Figure S8). This result would lend support to the conclusion that the reduction in the rescue observed in *clr1* strains is not caused by changes at *CEN3*, but likely reflect the effect of *CLR1* deletion. Taken together, these results suggest that overproduction of Cse4 can promote accurate segregation of *CEN*-inactive chromosomes, at least partly through *CLR* formation. The selective pressure caused by *CEN* inactivation might enhance the centromeric activity of *CEN*-proximal *CLR*s. The increased presence of kinetochore proteins around *CEN*s upon Cse4 OP may also contribute to the rescue phenotype [13].

Ndc10 is a budding yeast-specific essential kinetochore component required for the centromeric localization of many proteins, including Cse4 [27], [28]. We asked whether the rescue in segregation of *CEN*-inactive chromosome 3 observed in Cse4 OP strains is dependent on Ndc10. The GFP dots assay on a single nuclear division was repeated in WT and Cse4 OP strains when *CEN3* is inactivated, but now including the temperature-sensitive, conditionally-lethal *ndc10-1* allele, well known to abolish centromere function and cause chromosome missegregation [29]. The rescue in accurate chromosome segregation of *CEN*-inactivated chromosome 3 previously observed in Cse4 OP compared to WT disappeared (Figure 3E), as levels of accurate chromosome segregation in these strains became indistinguishable in the presence of *ndc10-1*. Combining this result highlighting the dependence on Ndc10 for the rescue observed in Cse4 OP with the previous results suggesting that this rescue is at least in part dependent on *CLR1* function (*clr1* strains), the assembly of functional *CLR*s might share some similarities to that of native *S. cerevisiae* centromeres, such as the functional requirement on Ndc10. This is also supported by the significant binding of Ndc10 at *CLR*s determined by ChIP experiments.

### *CLR*s share characteristics of both point and regional centromeres

**Figure 4:**
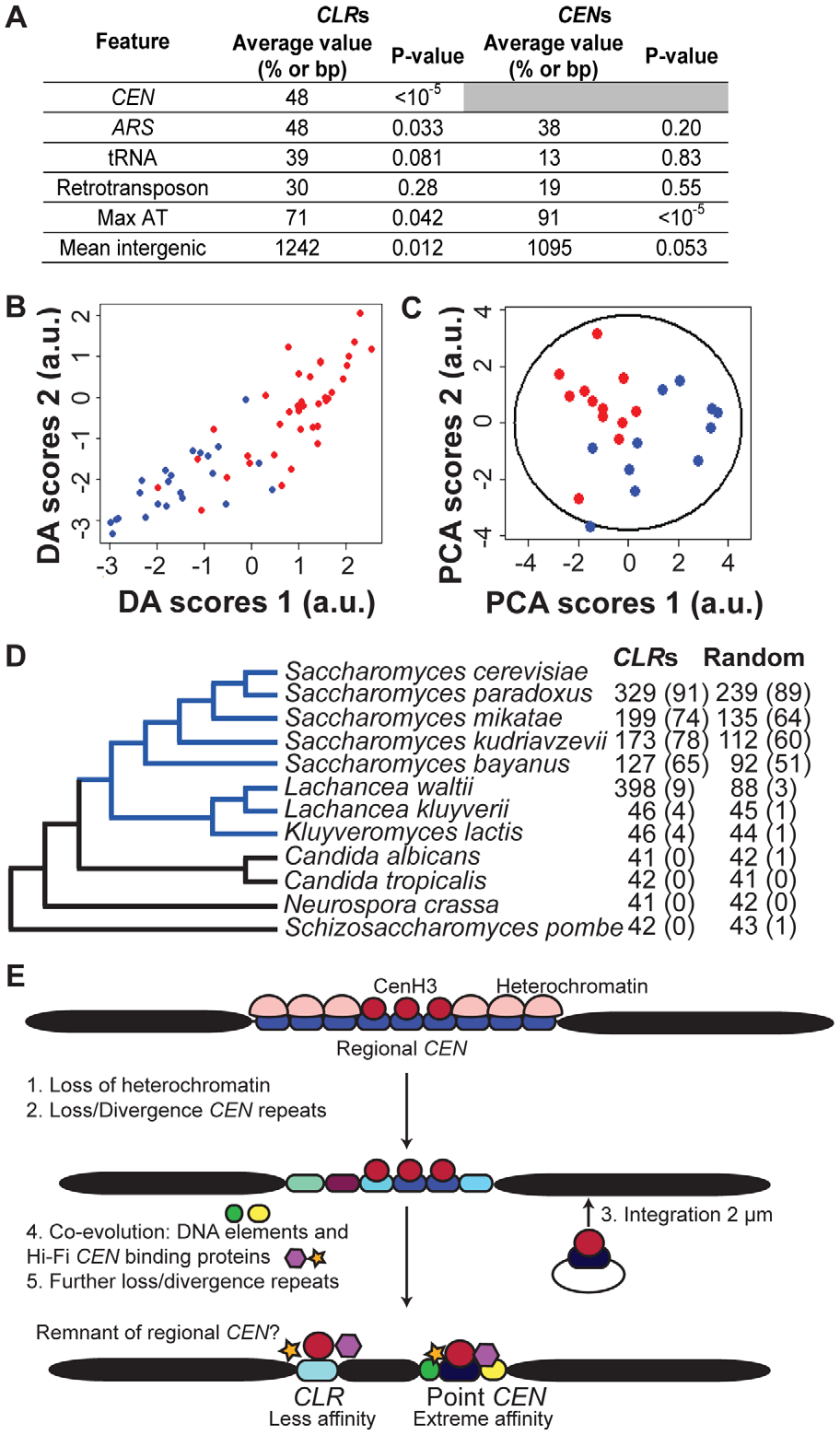
Genomic features of *CLR*s. (A) Comparison of *CLR*s and *CEN*s. Average value refers to the fraction of *CLR*s or *CEN*s located within 25 kb of a *CEN* or within 5 kb of an *ARS*, tRNA or retrotransposon. Mean intergenic indicates the average length of intergenic regions. Max AT represents the mean AT content of the most AT-rich 90-bp stretch of DNA. (B) *CLR*s can be separated from other genomic regions, using Discriminant Analysis (DA). Scores of *CLR*s (blue) and negative control regions (red) are plotted according to their discriminant function scores (Table 2). (C) Centromere proximity is a major contributor to variability among *CLR*s, as revealed by Principal Component Analysis (PCA). Scores of *CEN*-proximal (<25 kb, blue) and *CEN*-distal (>25 kb, red) *CLR*s are plotted relative to the first and second principal components (Table 3), along with a 95% confidence ellipse. (D) Conservation of *CLR* sequences among organisms with point *CEN*s (blue), but not with fungi bearing regional *CEN*s (black). Nucleotide blast (Blastn) was performed for 23 *CLR*s and 160 random intergenic regions. Mean BLAST scores are reported, with the percent of hits with a score over 45 (E<0.05) in parentheses. (E) Given our data and the confinement of *CLR* sequences to budding yeast bearing point centromeres, we proposed a modified version of the current model of centromere evolution (originally postulated in [1]), from regional to point *CEN*s, to account for *CLR*s. *CLR*s would represent evolutionary remnants from regional *CEN*s. Some AT-rich *CEN* repeats would have diverged but still retained the ability to bind Cse4 and other kinetochore proteins weakly, giving rise to the low-affinity *CLR*s observed in this study.

How do *CLR*s compare to native *S. cerevisiae CEN*s with regard to DNA sequence? Searching across all 23 identified *CLR*s for sequences similar to the centromeric *CDEI* or *CDEIII* consensus motifs did not yield clear results, nor did we find any motifs enriched amongst *CLR* sequences. *CLR* sequences tend to encompass a significantly AT-enriched, 90-bp stretch of DNA (Figure 4A; P = 0.042; MCMC simulation), reminiscent of the highly AT-rich *CDEII* element [30]. *CDEII* is the site where Cse4 binds [3], and it shares similarities to the alpha-satellite DNA repeats in the regional *CEN*s of higher eukaryotes [30]. Cse4 is essential for segregation of the multicopy 2 µm plasmid endogenous to yeast; this plasmid lacks a centromere and instead relies on Cse4 association with an AT-rich partitioning locus known as *STB* [31]. A chromosomally-integrated *STB* can also recruit Cse4 [31].

Which genomic characteristics best describe *CLR*s? In addition to their proximity to *CEN*s (Figure 4A; P<10^−5^; randomization tests), *CLR*s are often associated with *ARS*s, but not with tRNAs or retrotransposons (Figure 4A; randomization tests). They are also found in larger than average intergenic regions (Figure 4A; P = 0.012; randomization tests). These genomic features parallel those common at regional centromeres and neocentromeres of other yeasts. In particular, neo*CEN*s in *C. albicans* (activated by deletion of a native *CEN*) form mostly in large intergenic regions, and they are closely associated with replication origins [32], [33]. Gene ontology analysis of genes closest to *CLR*s did not reveal any enrichment for genes involved in particular cellular process (P<0.01).

### Non-centromeric Cse4 binding marks a subset of open chromatin sites

In Cse4 OP cells, fewer than 2% of non-centromeric Cse4 sites are also bound by all of the other three kinetochore proteins. To characterize non-centromeric Cse4 regions (both *CLR* and non-*CLR* regions), we compared Cse4 binding profiles to the genome-wide distribution of RNA polymerase II and Sono-Seq regions. Sono-Seq regions correspond to sites of highly-accessible chromatin [34]. Cse4 is incorporated mostly at intergenic and promoter regions (95% of Cse4 sites), in particular open chromatin (Figure S9). Cse4 binding is also correlated with overlapping or adjacent RNA polymerase II occupancy (Figure S9; Spearman's ρ = 0.32; P<10^−8^). Cse4 has been shown to undergo proteolysis to ensure physiological levels and protect from extensive stable euchromatinization with deleterious effects [35]. Comparing strains overproducing the more stable Cse4 allele Cse4^K16R^, subject to reduced degradation [35], to strains overproducing normal Cse4, we asked whether non-degradable Cse4 binds preferentially at *CLR*s. From ChIP-qPCR analyses, Cse4^K16R^ shows increased localization to the previously-tested *CLR*s (Figure 2B and Figure S3B) and to a set of non-*CLR* Cse4 binding sites located in gene promoters, but this enrichment occurs at similar levels in both *CLR*s and non-*CLR* Cse4 binding regions (Figure S10). Consistent with our finding that only a minority of Cse4 binding sites in Cse4 OP form *CLR*s, these results suggest that increased Cse4 retention alone does not appear to be the only determining factor in *CLR* formation.

Scm3 (HJURP) is a *CEN*-associated chaperone essential for cell viability [36]. Deletion of *SCM3* is suppressed by Cse4 OP [12]. By comparing Cse4 ChIP-Seq profiles in the presence or absence of *SCM3*, we found that Cse4 binding sites in both genotypes were highly concordant (Figure S11; Spearman's ρ = 0.85; P<10^−15^), suggesting that non-centromeric localization of Cse4 does not require Scm3. This finding is supported by the fact that transient incorporation of Cse4 on non-centromeric sites occurs at regions of high histone turnover, linking this phenomenon with nucleosome incorporation and ejection dynamics [37]. Cse4-containing nucleosome physical structure might also provide some potential reasons for the Scm3-independent Cse4 incorporation at non-centromeric loci. From *in vitro* reconstitution of Cse4-containing nucleosomes, two distinct populations of Cse4 nucleosomes have been reconstituted: one resembling canonical octameric nucleosomes and another found primarily at t AT-rich DNA characteristic of *CEN*s, atypical in its inclusion of Scm3 [38]. The former might predominate throughout the genome, while the latter would be highly specific for centromeres [39].

### Genomic context influences *CLR* formation

Twelve different variables (Table S1) were examined in an effort to find factors that distinguish *CLR*s from a control group consisting of regions that did not pass statistical filters set during ChIP-Seq analysis (Table 1); these control regions are referred to as *LCNCRs* (low-confidence, negative control regions). *CLR*s differed from the control group globally and across three individual variables: distance from *CEN*s, overlap with an intergenic region, and nearby RNA polymerase II occupancy (P<0.05; MANOVA; ANOVA). Group membership of individual sites could be predicted quite accurately using discriminant analysis or k-means clustering (78% and 83% success, respectively; Figure 4B and Table 2). As determined by discriminant analysis, we found that *CLR*s are closer to *CEN*s, have a more AT-enriched 90-bp stretch of DNA, and are located in larger than average intergenic regions, with lower transcription at nearby genes, in comparison to the control group (Figure 4B and Table 2). This difference between *CLR*s and *LCNCR*s is quite apparent from the discriminant function plot, with low mixing between groups (Figure 4B). There is, however, some variation among *CLRs* with respect to distance from *CEN*s, AT content and the presence of nearby open chromatin, as revealed by principal component analysis (Figure 4C and Table 3). *CEN*-distal and *CEN*-proximal *CLR*s form somewhat separated groups on the principal component score plot, and sites within each group tend to cluster together, as is particularly evident for *CEN*-distal *CLR*s (Figure 4C). In addition, when performing a discriminant analysis among the *CLR*s themselves according to their position in the ranked target list (Table 1; group 1 (*CLR1–12*) vs. group 2 (*CLR13–23*)), we observed that the strongest discriminant was the distance to *CEN*s, with *CLR*s in the top tier usually closer to *CEN*s. This is consistent with our previous observations from the ChIP-Seq data. The proximity of *CLR*s to centromeres suggests that pericentric chromatin creates a preferred environment for establishment of *CLR*s [14]. Alternatively, centromere-distal *CLR*s may be disfavored due to a greater risk for instability, in the same way that increased distance between *CEN*s in dicentric chromosomes increases instability [40]. These results strongly suggest that chromatin structure and chromosomal context play roles in *CLR* formation.

**Table 2:** Results from linear discriminant analysis of 23 *CLR*s and 38 negative control regions (*LCNCR*s).

Variable	Discriminant coefficient^1^
**Percent of binding site length overlapping an ORF**	1.006
**Mean AT content**	0.988
**Distance to ** ***CEN***	0.764
**Maximum AT content**	−0.758
**Length of closest intergenic region**	−0.482
Distance to retrotransposon	−0.421
Rank closest Sono-Seq region, scored against HA control IP	0.322
Distance to tRNA	0.269
Rank closest Sono-Seq region, scored against Myc control IP	−0.225
Rank closest PolII region	−0.198
Distance to *ARS*	0.104
Rank closest Cse4 region, in a Cse4 WT strain	−0.043

Variable	Discriminant coefficient^2^
**Maximum AT content**	−1.231
**Mean AT content**	1.159
**Distance to ** ***CEN***	0.666
**Rank closest PolII region**	−0.591
**Length of closest intergenic region**	−0.546
Rank closest Sono-Seq region, scored against HA control IP	0.280
Distance to retrotransposon	−0.187
Rank closest Cse4 region, in a Cse4 WT strain	−0.125
Distance to tRNA	0.107
Rank closest Sono-Seq region, scored against Myc control IP	−0.032
Distance to *ARS*	0.017

Discriminant variables and their coefficients are shown, with the top 5 discriminant variables in bold. Raw data are given in Table S1.

1 Discriminant analysis including all 12 variables.

2 Discriminant analysis with 11 variables, excluding the variable “percent of length of binding site overlapping an open reading frame (ORF)”. Refer to the Materials and Methods section.

**Table 3:** Results from principal component analysis of 23 *CLR*s.

First principal component		Second principal component		Third principal component	
Variable	Loading	Variable	Loading	Variable	Loading
Rank closest Sono-Seq region, scored against Myc control IP	0.451	Percent of binding site length overlapping an ORF	−0.460	Distance to retrotransposon	0.588
Rank closest Sono-Seq region, scored against HA control IP	0.446	Rank closest PolII region	0.457	Distance to tRNA	0.577
Mean AT content	0.436	Rank closest Cse4 region, in a Cse4 WT strain	0.403	Rank closest Sono-Seq region, scored against HA control IP	0.303
Maximum AT content	0.372	Length of closest intergenic region	0.373	Length of closest intergenic region	−0.295
Distance to *CEN*	−0.353	Distance to *CEN*	0.293		

Three components were significant according to parallel analysis. Only variables with loadings above |0.250| are presented. Raw data are given in Table S1.

### Conservation of *CLR* sequences and chromosomal context elements

*CLR*-containing regions are conserved among the *Saccharomyces* lineage and other closely-related budding yeasts with point *CEN*s [41], but not with more divergent fungi (Figure 4D; blastn). In general, *CLR* sequences are more conserved than a randomly-selected set of intergenic regions (Figure 4D; P<0.05 across *Saccharomyces sensu stricto*; MCMC simulation of blastn scores). Although the role of *CLR*s in wild populations of yeasts remains unknown, sequence conservation with other budding yeasts suggests that *CLR*s possess a conserved function.

With the development of the new *Saccharomyces sensu stricto* database [42], it is possible to analyse some of the genomic characteristics and chromosomal context features, including the association to *CEN*s, the association to tRNAs, the mean length of intergenic regions, and the mean AT content in the most AT-enriched 90-bp stretch of DNA, in closely-related budding yeast. Trends similar to those observed for *S. cerevisiae CLR*s were observed for sequences similar to *CLR*s present in *S. mikatae*, *S. kudriavzevii* and *S. bayanus* (Figure S12). Proximity to *CEN*s was even more striking in those three fungi than in *S. cerevisiae* (Figure S12A). In the three non-*S. cerevisiae* fungi analyzed, *CLR*-related sequences were also present in larger than average intergenic regions and encompassed a significantly AT-enriched, 90-bp stretch of DNA (Figure S12). The only difference concerned association with tRNAs: while *CLR* association to tRNAs was only marginally significant in *S. cerevisiae* and *S. bayanus*, it was significant in *S. mikatae* and *S. kudriavzevii*, although very close to P<0.05 significance threshold. In addition to primary sequence conservation, these results highlight the conservation of genomic features and chromosomal context elements associated with *CLR*s in closely-related budding yeasts.

Overall, *CLR*s share many features with both regional and point centromeres. Like regional *CEN*s, *CLR*s are not entirely sequence-defined; rather, they are defined largely by features pertaining to chromosomal context. Like point *CEN*s, they are rather small (<1 kb) and contain a short *CDEII*-like, AT-rich stretch of DNA. According to previous models of centromere evolution, an epigenetic regional centromere evolved to point centromeres in a few steps [1]. First, heterochromatin would be loss. Then, *CEN* repeats would diverge and/or disappear. Third, a segregation locus from a self-propagating genetic element (such as the *STB* locus in the yeast 2 µm plasmid) would integrate on the chromosome, with the potential to successfully hijack the segregation machinery. Once this is accomplished, it is likely that *CEN* repeats would diverge or disappear even more. Finally, acquisition of specific DNA modules and evolution of segregation proteins that bind this newly-integrated locus would create point centromeres with high specificity. If this model is correct, then *CLR*s may be remnants of regional *CEN*s that were lost or diverged during evolution (Figure 4E). *CLR*s might resemble divergent AT-rich *CEN* repeats, able to bind Cse4 and function as a strong centromere unit in the past, that still retained some ability to recruit Cse4, and other kinetochore proteins weakly. This is the model most supported by parsimony and by our evolutionary analyses. Indeed, using a bioinformatics approach, we identified *CLR* sequences only in budding yeast bearing point *CEN*s, not in those carrying regional *CEN*s. In general, centromeric building blocks with weak activity, such as an individual *CEN* repeat, a plasmid element or a short sequence similar to a *CLR*, might have been rendered more efficient through their massive multimerization (regional *CEN*s) or, as in the *S. cerevisiae* lineage, through the acquisition of specific DNA modules (point *CEN*s and *STB*) to form stable, strong centromeres with high segregation fidelity.

*CLR* formation affects chromosome segregation differently, depending on whether the chromosome has an active centromere or not. Recent data from an assay measuring the transmission fidelity of an artificial chromosome indicated a significant, although modest, increase in chromosome loss (about 2 fold) when Cse4 is overproduced and the normal centromere is functional [43]. In Cse4 OP, when the *CEN* is active, the observed increase in chromosome instability is likely due to the formation of functional *CLR*s in a subset of the cell population. The modest effect is concordant with the lower levels of protein binding at *CLR*s vs. *CEN*s, and with the higher incidence of *CEN*-proximal *CLR*s. Based on studies of dicentric chromosomes, *CEN*-proximal *CLR*s have potentially less deleterious effects than *CEN*-distal *CLR*s [40]. In contrast, when the normal *CEN* is inactive, *CLR* formation promotes, at least partially, accurate chromosome segregation of the *CEN*-inactivated chromosome, which might be beneficial to yeast cells under this condition.

New *CEN*s have been created artificially through two mechanisms: tethering of outer kinetochore components onto DNA [17], and increased production of kinetochore proteins [44]. At the DNA level, strategies to generate centromere activity have ranged from deletion of a native *CEN* in *C. albicans* [33] to induction of chromosome rearrangements by radiation in flies and plants [45]. Interestingly, studies in *Drosophila* and barley have revealed a predisposition for neo*CEN*s to form in pericentric chromatin [45]. More recently, studies conducted on *in vitro* chromatin templates [46] and in cultured *Drosophila* cells [47] have reiterated the essential role of Cse4 and proven its sufficiency in the formation of kinetochores. Targeting Cse4 [46], [47] or Cse4-associated factors [48] directly onto chromosomes or plasmids can generate heritable centromeric activity. However, despite the deposition of Cse4 at a specific location, not all cells recruited kinetochore components and established a kinetochore, presumably due to chromatin effects [48]. Similarly, in the present study, we observed *CLR* formation only at a subset of all non-centromeric Cse4 binding sites.

New *CEN*s also occur naturally. In humans, acentric chromosomes resulting from chromosome rearrangements can be stabilized through the establishment of neocentromeres at sites devoid of α-satellite repeats and containing little or no heterochromatin [49]. The aneuploidy so often observed in cancer cells may arise from ectopic kinetochore formation and/or destabilization of native *CEN*s. Many liposarcomas carry a supernumerary chromosome containing oncogenes and a neo*CEN* [45]. Colorectal cancer cells exhibit overproduction of Cse4, which is mistargeted to non-centromeric loci [4]. Moreover, comparative genomics has identified latent *CEN*s that have been recurrently used throughout primate evolution [50]. *CLR*s, evolutionarily-new centromeres, ectopic neocentromeres and bacterial centromere-like regions are likely to provide additional insights into the origin, evolution, establishment and maintenance of native centromeres.

## Materials and Methods

### Strains and plasmids

Yeast strains are isogenic with W303 (Table S2). Cse4 is tagged internally with a 3HA epitope; this tagged version can act as the sole Cse4 copy in a haploid cell without deleterious effect [5]. All other proteins are tagged at their C-termini. The *ARS* plasmid (pPL26) was generated by cloning a 0.9 kb fragment containing *ARS1* into pRS305 [18]. *CLR* and *CEN6* sequences were integrated into pPL26. *ARS-CEN6* plasmid pRS315 was also used in this study.

### Chromatin immunoprecipation–sequencing (ChIP–Seq)

ChIP-Seq experiments were performed at least in independent biological duplicates, as described previously [9]. Yeast strains were grown in 500 mL YP media supplemented with adenine and uracil, in presence of glucose or galactose/raffinose, to mid-log phase (OD_600_ = 0.5–0.7). Proteins were crosslinked to DNA by treating cells with formaldehyde (1% final concentration) for 15 minutes, then quenched with glycine. Cells were collected by filtration after two washes. After cell lysis using a FastPrep machine (MP Biomedical), chromatin was sheared by sonication using a Branson Digital 450 sonifier (Branson). Clarified, sonicated lysates were taken at this step for Sono-Seq, prior to immunopreciptation [34], [51]. Immunoprecipitations of Myc-tagged and HA-tagged strains, as well as those of the respective control untagged strains, were carried out overnight with EZ-View anti-Myc or anti-HA affinity gels (Sigma). For native RNA Polymerase II ChIP, cell lysates were incubated with Pol II 8WG16 mouse monoclonal antibody (Covance) and pulled down using Protein G agarose beads (Millipore). After several washes and reversal of protein-DNA crosslinks, ChIP DNA was purified through a Qiagen MinElute PCR purification column (Qiagen). Illumina sequencing libraries were generated using adapters for multiplexing [9]. Four barcoded libraries were mixed in equimolar ratios and processed on an Illumina Genome Analyzer II. Each sequence read consisted of a 4-bp index and at least 26 bp from the sample. An average of 2.1 million uniquely mapping sequence reads per biological replicate was obtained, corresponding to an overall mapping of 56.2% to the S288c reference genome (SGD/UCSC sacCer2 version, June 2008). We have also used previously published Cse4 ChIP-Seq data deposited in the Gene Expression Omnibus (GEO) database under GSE13322 [5], [52]. Data generated from this study have been deposited in GEO under accession number GSE31466.

To collect ChIP samples of *mcd1-1* background, cultures were grown overnight at the permissive temperature (25°C) to OD_600_ = 0.3–0.4 and then shifted to the restrictive temperature (37°C) for ∼2.5 h prior to crosslinking, similarly to other protocols for the abrogation of the pericentric intramolecular C loop [14]. After ∼2.5 h, most cells (>95%) were large-budded.

To collect ChIP samples for the non-degradable Cse4 experiment [35], Myc-Cse4 and Myc-Cse4^K16R^ strains were grown in SC Raffinose/Galactose – URA overnight to OD_600_ = 0.3, overproducing normal and non-degradable Cse4 respectively. Cells were then transferred in rich media (YPAU+Raffinose/Galactose) and grown for ∼4 h to OD_600_ = 0.8–1.0, for easier comparison with our previous ChIP-qPCR data.

### Identification of *CLR*s

Raw sequencing data were first processed by Illumina's analysis pipeline. Reads were then parsed according to the index. Remaining bases were aligned against *S. cerevisiae* S288c reference genome version 2 (SGD/UCSC sacCer2) by the ELAND algorithm (Illumina). The peak scoring algorithm PeakSeq [10] was used to identify statistically significant binding sites. ChIP-Seq data from epitope-tagged strains were scored against ChIP-Seq data from their matching untagged strains. Scoring reference sets were created by pooling uniquely-mapping reads from biological replicates of untagged control strains. As a reference sample marking open chromatin [34], two lists of Sono-Seq regions were generated, scored against either anti-Myc or anti-HA control sets.

To uncover *CLR*s, we took a conservative, stringent approach to minimize false positives lacking functional significance or failing qPCR validation. For each biological replicate, only putative regions with q-value <10^−5^ were considered [11]. Binding sites from Cse4, Mif2, Ndc10 and Ndc80 ChIP-Seq data were overlapped (maxgap = 150). To identify a binding region as a *CLR*, 1) all four kinetochore proteins must be present given the q-value threshold; and 2) for proteins in direct contact with DNA, mean PeakSeq ratios between duplicates should be above 2.0 for open chromatin marker Cse4 and 1.5 for direct DNA binders Mif2 and Ndc10 [5], [53]. Several other filters were used to distinguish between low and high confidence regions for subsequent functional analyses, including comparison of PeakSeq experimental and background reads between *CLR*s and *CEN*s, inspection of normalized signal tracks (high tagged/untagged ratio and low background in untagged controls desired), binding over a highly PolII-occupied ORF, and presence in HOT regions [54]. 23 putative loci, termed *CLR*s, met these criteria in Cse4 OP and none in WT (Table 1). Included in the same Table are other sites (*LCNCRs*) that did not pass the aforementioned filters, used during computational analyses. For WT, only one low-confidence negative region, the rDNA array, was found after unmasking for repeated regions. GO analysis for this control group showed a significant enrichment for metabolic genes.

Significant binding regions for RNA Polymerase II and Sono-Seq were determined using q<10^−5^ and PeakSeq ratio ≥2.00.

### Real-time quantitative PCR validation of *CLR*s

Real-time quantitative PCR (qPCR) was performed to validate the presence of kinetochore components Cse4, Mif2, Ndc10 and Ndc80 at six *CLR*s. These six *CLR*s were randomly selected and spanned multiple confidence levels of our final ranked target list (one at the top, two in the middle, and three at the bottom) [55], [56]. As a positive control for ChIP experiments, we monitored binding of these four proteins at a native centromere. Two negative primer pairs were used for accurate determination of enrichment values. Primers were designed using Primer3 (http://frodo.wi.mit.edu/primer3/) and primer sequences are given in the Table S3. qPCR reactions were set up in triplicates with SYBR green dye and run on a Roche LightCycler480 according to the manufacturer's recommendations, using the same amplification program as previously described [5]. Each primer pair was tested on a dilution series of yeast genomic DNA to determine its efficiency. For every primer pair, a single PCR product was amplified, given the presence of a single peak in melting curve analyses. The “Second derivative maximum” analytical tool in the Roche LightCycler480 was used to obtain Crossing point values (C_p_). Enrichments were calculated by the 2^−ΔΔCp^ method [57]. First, for any given primer pair, a raw ratio between experimental samples (Myc- or HA-tagged strains) and control samples (untagged strains of similar genotype immunopreciptated with anti-Myc or anti-HA antibodies) was obtained. Then this raw ratio for a positive primer pair was divided by the raw ratio found for a control, negative primer pair, resulting in a normalized enrichment value. Enrichment values were averaged for all biological replicates, with the appropriate standard errors of the mean.

### Signal tracks

Signal track files were visualized in the Integrated Genome Browser, with y-axes scaled according to the number of uniquely-mapped reads and with annotations from the *Saccharomyces* Genome Database.

### Target list annotation, target list agreement, and Gene Ontology analysis

Target lists from different biological replicates were merged and annotated to find overlapping and/or nearest genomic features using various R and Bioconductor packages, mostly ChIPpeakAnno [58], and also biomaRt, coda, lattice, MASS, rjags, seqinr and stats packages.

Target lists were first sorted by q-value and then by the difference between PeakSeq experimental and background reads. Pairwise comparisons of lists were done using ChIPpeakAnno with parameters maxgap = 0 and multiple = T. Spearman's rank correlation coefficients and associated p-values for overlapping peaks were computed.

GO Biological Process Ontology analyses (p-value <0.01) were performed on SGD's website. We compared GO results from *CLR*s and *LCNCR*s. GO analysis for *CLR*s did not give any significant term. GO analysis for *LCNCR*s showed a significant enrichment for metabolic genes (data not shown).

### Western blotting

Whole-cell protein extracts were obtained using the post-alcaline yeast protein extraction method [59], for 4 biological replicates. Briefly, for WT and Cse4 OP strains, 2 mL of yeast culture at OD_600_ = 0.8 were isolated, medium was removed and cells were frozen at −80°C. Cells were resuspended in water, an equal volume of 0.2 M NaOH was added and samples were incubated at room temperature for 5 min., after which NaOH was removed thoroughly. Samples were then boiled in 1× sample buffer containing 5% β-mercaptoethanol for 4 min. and the supernatant was kept. Samples were run for protein gel electrophoresis on a 4–12% Novex NuPAGE Bis-Tris gel (Invitrogen) in MOPS buffer. Proteins were then transferred a PDVF membrane on a semi-dry Trans-Blot SD apparatus (BioRad). Membranes were blocked with TBS+0.1% Tween with 5% dry milk. Primary antibodies were added for an overnight incubation: mouse anti-HA 12CA5 and mouse anti-β-actin (Abcam). After washes in 1× TBS+0.1% Tween, a HRP-conjugated anti-mouse IgG secondary antibody was added in 1× TBS+0.1% Tween with 5% dry milk for 1.5 h. Following washes in 1× TBS/T, the SuperSignal West Pico Chemiluminescent Substrate (Thermo) was added to the blot and detection was done on a STORM imager (GE Healthcare). Western blot images were processed and analyzed using the ImageQuant software (GE Healthcare). Cse4-3HA protein levels were normalized by the abundance of β-actin in each replicate.

### Plasmid stability assays

Plasmid assays to test *CLR* sequences inserted into an *ARS* plasmid were conducted according to standard procedures [2], [18]. For all plasmid analyses, at least six different transformants were grown. Given that we observed some variability in plasmid assays, we used fluctuation analyses for each data point, taking the median value of 3–5 technical replicates from a single transformant as one data point [60].

For doubling time analyses, cells were grown overnight in synthetic complete (SC) media lacking leucine (SC-Leu), with raffinose and galactose as carbon source. Cultures were diluted around 5×10^6^ cells in the same media. Optical densities were measured every 2–4 hours. Doubling times were calculated in R. Statistical significance was tested by a Bayesian analysis with Markov Chain Monte Carlo (MCMC), using R package rjags (JAGS, http://www-ice.iarc.fr/~martyn/software/jags/). MCMC simulations let the data and its variability generate sampling distributions of the maximum likelihood estimator without strong prior or test assumptions; p-values were calculated from 100,000 comparisons of this estimator between 2 groups.

For plasmid retention and colony formation analyses, cells were grown for ∼4 generations in rich medium, with raffinose and galactose as carbon sources. Cultures were diluted 10-fold or 100-fold and plated on SC-Ade-Leu and SC-Ade plates. Photos were taken after 4 days of growth. Pictures of transformation plates on SC-Ade-Leu with galactose/raffinose also represent 4 days of growth after transformation.

### Chromosome segregation assays

Chromosome segregation analysis of GFP dots present on chromosome 3 was performed in biological triplicates as described [17], without major modifications. Briefly, cells were grown overnight to early log phase in rich medium containing raffinose, and alpha factor was then added to a final concentration of 10 µg/mL. Following a 2 h incubation at 25°C, cultures were resuspended in YPAU with raffinose/galactose or raffinose only, still in presence of alpha factor, and then placed at 37°C for 1 h. Next, cells were washed 4 times in the same media, pre-warmed at 37°C and devoid of alpha-factor, and released at 37°C for about 5 h to accumulate populations in which most cells were in telophase due to the *cdc15-2* allele. Experiments including the additional temperature-sensitive allele *ndc10-1* were performed similarly. About 98% of cells were large-budded. GFP dots were visualized in live cells and classified into two categories: 1) one GFP dot in each cell, or 2) two GFP dots in the same cell. A minimum of 200 cells were counted per replicate. Statistical significance was assessed using Fisher's exact test. In addition, for each sample, an aliquot was quick-fixed with ethanol and DAPI was added. >90% of cells had segregated DNA between their buds, as revealed by DAPI staining. Experiments plotted in Figure 3E were from cultures resuspended in raffinose/galactose, containing a conditional *CEN3* (OFF) or not (ON). Strains comprising conditional *CEN3* were also resuspended in raffinose-only media (ON) and gave similar high percentages of cells with accurate segregation. Overnight growth of cells in raffinose-only media, with an active conditional *CEN3* (ON), yielded >99% of cells with accurate segregation.

Single-cell pedigree analysis was performed on strains containing a conditional centromere [19], [25]. Cells were grown to early log phase in YPAU+raffinose. Galactose was added to the liquid medium for ∼30 min. (final concentration 1%), prior to plating on a YPAU+galactose/raffinose plate. Unbudded cells were isolated, and plates were incubated for 2–3 hours. Daughter cells were separated from their mothers and monitored for bud formation as a function of time. Statistical significance was assessed using Fisher's exact test. To ensure proper timing of cell divisions in Figure 3D and in Figure S7, cells containing a conditional *CEN3* (OFF) or not (ON) were plated on galactose/raffinose plates. Strains comprising conditional *CEN3* were also plated on YPAU+dextrose plates (ON) and gave end-point results comparable to those of Figure 3D, with >90% of budded daughter cells.

### Statistical significance of *CLR* association

We determined the number of *CLR*s located within 5 kb of tRNAs, ARSes or retrotransposons. 23 sites were randomly chosen on chromosomes containing a putative *CLR*. The number of chosen sites on a chromosome paralleled the chromosomal distribution of *CLR*s. The number of random sites falling within 5 kb of a feature was determined for 100,000 iterations. The p-value is given by the fraction of iterations with greater or equal feature association than found across *CLR*s. For these discrete genomic features, we adjusted p-values using a Bonferroni correction.

For centromere proximity, a similar procedure was followed. A site is centromere-proximal if located within 25 kb of the centromere.

For association tests performed in other fungi than *S. cerevisiae*, the number of random sites chosen followed the number of *CLR* sequences deemed conserved by blastn in each species (Figure 4A): 17 in *S. mikatae*, 18 in *S. kudriavzevii* and 15 in *S. bayanus*. Sequence annotation data were obtained from the *Saccharomyces sensu stricto* database [42].

### Statistical significance of the presence of *CLR*s in larger than average intergenic regions

We considered the region comprised between two ORFs as the intergenic region. The mean length of intergenic regions encompassing a *CLR* was determined, excluding two *CLR*s that partly overlapped putative ORFs. 21 intergenic regions were randomly selected 100,000 times. For any iteration, the mean length was computed and compared to the actual value. P-values correspond to the fraction of iterations with a greater or equal mean length.

In other fungi than *S. cerevisiae*, the number of random intergenic regions chosen followed the number of *CLR* sequences found in intergenic regions and deemed conserved by blastn in each species (Figure 4A): 17 in *S. mikatae*, 18 in *S. kudriavzevii* and 15 in *S. bayanus*. Sequence annotation data were obtained from the *Saccharomyces sensu stricto* database [42].

### Signal aggregation plots around centromeres

For each protein and for untagged controls, we determined the number of uniquely mapped reads, per million mapped reads, at every nucleotide position in a 4-kb region centered in the middle of the centromere. Values for each protein were averaged to generate a mean kinetochore protein signal. Log ratios between this signal and the control signal were plotted. Aggregation plots around *CEN2* and *CEN5* for individual proteins are given in Figures S13 and S14 respectively.

To test the significance of the increased broadness of kinetochore signal seen at centromeres, we calculated the peak width at each centromere. Width was determined as the length of centromeric signal where the ratio between the mean kinetochore protein signal and the control signal was ≥2. A paired t-test, comparing each *CEN* between WT and Cse4 OP, was performed.

### Maximum AT content

For 23 *CLR*s and 38 *LCNCR*s, a 90-bp window was slid to determine the maximum AT content in a 500-bp region, centered at the genomic location of the average kinetochore protein signal maximum, keeping the percentage of A and T in the most AT-rich, 90-bp stretch. Maximal values from *CLR*s and *LCNCR*s were compared for statistically-significant differences by MCMC simulations. A similar procedure was used for other fungi than *S. cerevisiae*, with sequence data obtained from the *Saccharomyces sensu stricto* database [42].

### *CLR* sequence comparison across fungal genomes

For each *CLR*, a 400-bp sequence, centered at the genomic location of the average kinetochore protein signal maximum, was considered for evolutionary analyses. Inspection of the selected regions was carried out to ensure that the sequences did not contain repetitive and/or highly conserved features, such as a Ty element or a well-characterized ORF, if possible. Nucleotide BLAST (Blastn) was performed on genomes deposited at NCBI (NCBI's Fungal Genomes BLAST page, http://www.ncbi.nlm.nih.gov/sutils/genom_table.cgi?organism=fungi) with the following parameters: expect value (E) <1, and other values as default [61].

A phylogenetic tree indicates, for each species, the fraction of *CLR*s with at least one significant hit (score of 45 or higher, E<0.05) and the average hit score across all *CLR*s.

We compared *CLR* values with 160 randomly-selected intergenic regions of same length to determine whether sequence conservation is greater in *CLR*s. Blastn was carried out as described above for this random set. Statistical significance of average blastn scores was tested by MCMC simulations.

### Principal component and discriminant analyses

For 23 *CLR*s and 38 *LCNCR*s, data from 12 variables were obtained (Table S1). Individual variables were either 1) left untransformed, 2) log-transformed, or 3) square root-transformed to normality or near-normality as visualized by quantile-quantile normal plots. Data from all 61 sites, or from each group, followed a multivariate normal distribution (Figure S15). On multivariate χ^2^ plots, all data points lie within the 95% confidence intervals of multivariate normality (Figure S15).

Principal component analysis was performed for the 23 high-confidence *CLR*s only, with data standardized by the correlation matrix, in R. Principal component analysis gives the direction of most variability to spread out data points and determine variables and sites that behave similarly. The number of significant principal components was determined by parallel analysis. Principal component score plots were generated using the first (x-axis) and second (y-axis) principal components. A 95% confidence ellipsis was added to the score plots.

Linear discriminant analyses between *CLR*s and *LCNCR*s were performed on standardized, scaled data in R, to identify variables that would distinguish these two well-defined groups. When all 12 variables were present, the percentage of a binding site overlapping an ORF was a very strong discriminator but could be perceived as arbitrary, depending on the length of the binding site. Therefore discriminant analysis was also performed with all variables except that one. Discriminant score plot was generated using the discriminant function with all variables (x-axis) and the discriminant function with all variables except percent overlap with an ORF (y-axis). We used stepwise discriminant analysis to determine the variables that discriminate best between groups, in SAS, and also obtained similar results. Overall discriminative power was tested using cross-validation (leave-one-out classification). As a comparison, k-nearest neighbors classification was performed on the same scaled, standardized dataset, using k = 3.

## Supporting Information

Figure S1 Western blot analysis of Cse4 levels in WT and Cse4 OP strains. (A) Western blot image showing the levels of Cse4-3HA (internal tag) in Cse4 OP and WT strains, as well as β-actin as a loading control. (B) Quantitation of Cse4 protein levels in WT and Cse4 OP strains. Cse4 abundance was normalized by the β-actin protein levels. Normalized Cse4 levels (means in arbitrary units (a.u.)+/−standard errors of the mean (SEM)) are plotted on a linear scale. Individual enrichments were obtained from four biological replicates.
(TIF)

Figure S2 All *CEN*s are occupied by four kinetochore proteins, in WT and Cse4 OP strains. ChIP-Seq signal tracks for Cse4 (red), Mif2 (blue), Ndc10 (green) and Ndc80 (orange) are scaled according to the number of uniquely-mapping reads, in WT (A) and Cse4 OP strains (B). Significant binding sites are represented by a liked-colored box under its corresponding signal track. Control samples (immunoprecipitates from untagged strains) are shown in grey. Open reading frames (ORFs) are represented by purple boxes. The black circles indicate centromeres. Horizontal scale bars represent 1 kb.
(TIF)

Figure S3 ChIP-qPCR validation of 6 *CLR*s. (A) All tested *CLR*s were not bound by all four proteins in WT. Only Cse4 in *CLR*1 and Ndc10 in *CLR7* displayed significant binding (normalized enrichment ratio >2, dotted line). (B) In Cse4 OP, significantly enriched protein binding was confirmed at the six *CLR*s tested, for all four proteins examined (normalized enrichment ratio >2, dotted line). Normalized enrichment ratios (means in arbitrary units (a.u.)+/−standard errors of the mean (SEM)) are plotted on a linear scale. Individual enrichments were obtained from qPCR reactions run in triplicates and performed in at least two biological replicates. Note the different scales between (A) and (B). A normalized enrichment of 1 indicates no enrichment over a negative control region not enriched for kinetochore proteins.
(TIF)

Figure S4 Kinetochore proteins are present at *CLR*s when the pericentric intramolecular C loop is abrogated in a cohesin-deficient *mcd1-1* background. (A) All tested *CLR*s were not bound by all four proteins in strains with normal Cse4 levels (WT) in a *mcd1-1* background, similarly to Figure S3A. Only Cse4 in *CLR*1 and Ndc10 in *CLR7* displayed significant binding (normalized enrichment ratio >2, dotted line). (B) In strains with elevated Cse4 levels (Cse4 OP) in a *mcd1-1* background, significantly enriched protein binding was confirmed at the six *CLR*s tested, for all four proteins examined (normalized enrichment ratio >2, dotted line), similarly to Figure S3B. Normalized enrichment ratios (means in arbitrary units (a.u.)+/−standard errors of the mean (SEM)) are plotted on a linear scale. Individual enrichments were obtained from qPCR reactions run in triplicates and performed in at least two biological replicates. Note the different scales between (A) and (B). A normalized enrichment of 1 indicates no enrichment over a negative control region not enriched for kinetochore proteins.
(TIF)

Figure S5 Colony formation on plates from plating assays. Strains bearing *CLR* plasmids generate colonies of intermediate size, which are on average larger than those carrying *ARS* plasmids and smaller than those containing *CEN* plasmids. After a few generations of growth in non-selective rich medium, strains carrying various plasmids were plated on selective medium (-Ade -Leu) for four days. Strains plated on permissive medium (-Ade) do not differ in colony size.
(TIF)

Figure S6 Comparison of *ARS-CLR* plasmids and *ARS* plasmids bearing random inserts of similar sizes in plasmid segregation assays. (A) Doubling times in selective medium of strains carrying different plasmids (Means+/−SEM). (B) Fraction of plasmid-containing cells after growth in non-selective media for strains bearing various plasmids (Means+/−SEM). P-values were computed using MCMC simulations (* p<0.05, ** p<0.01, *** p<0.001,. p<0.10) (A–B). *ARS-R1* and *ARS-R2* refer to *ARS* plasmids bearing random inserts of 1 kb and 0.8 kb, respectively.
(TIF)

Figure S7 Time course analysis of daughter cell budding after transfer of mother cells to a galactose plate. Values (Means+/−SEM) are given for various genotypes at 3-h intervals (refer to Figure 3D). ON and OFF indicate the presence of the conditional *CEN3*.
(TIF)

Figure S8 Binding levels of kinetochore proteins at *CEN3* are similar in *clr1* and *CLR1+* strains. ChIP-qPCR confirms that the presence of kinetochore proteins at *CEN3* is not affected when *CLR1* is deleted. Individual protein enrichments at *CEN3* were normalized and compared in strains containing *CLR1* (*CLR1+*) and missing *CLR1* (*clr1*), for WT (A) and Cse4 OP (B). Normalized enrichment ratios (means in arbitrary units (a.u.)+/−SEM) were plotted on a log 10 scale. A normalized enrichment of 1 indicates no enrichment over a negative control region not enriched for kinetochore proteins.
(TIF)

Figure S9 Cse4 marks a subset of open chromatin. Cse4 is associated with promoters, accessible chromatin and RNA polymerase II-bound regions. Cse4 shows a broader euchromatin distribution upon its overproduction, consistent with previous reports [62]. (A) On chromosome 9, regions of Cse4 (red) binding overlap promoters, regions bound by RNA polymerase II (blue) and Sono-Seq (green) sites. Sono-Seq regions are enriched for open chromatin [34], [51]. Promoter nucleosomes and regions of high histone turnover have been associated with higher levels of non-centromeric Cse4 [37], [63]. Cse4 binding is also correlated with overlapping or adjacent RNA polymerase II occupancy (Spearman's rho = 0.32; P<10^−8^), in concordance with the presence of Cse4 around transcribed regions [5], [12]. (B–C) An extra-centromeric Cse4 binding region most commonly overlaps open chromatin (i.e. promoters and Sono-Seq region), and is adjacent to an ORF bound by RNA polymerase II. Examples on chromosomes 8 (B) and 9 (C) are shown. Control samples (immunoprecipitates from untagged strains) are shown in grey. Open reading frames (ORFs) are represented by purple boxes. Horizontal scale bars represent 1 kb. Significant regions of protein binding or sensitivity to Sono-Seq are represented by a like-colored box under the corresponding signal tracks.
(TIF)

Figure S10 Non-degradable Cse4 is not preferentially enriched at *CLR*s compared to non-*CLR* Cse4 binding sites at gene promoters. (A) ChIP-qPCR comparative analyses of *CLR*s and non-*CLR* promoters bound by Cse4 indicate that non-degradable Cse4^K16R^ is relatively more abundant than normal Cse4 at both *CLR*s and non-*CLR* Cse4 binding sites, in similar proportions. Non-degradable Cse4 enrichments for 6 *CLR*s (same as Figure 2 and Figure S3) and for 6 non-*CLR* Cse4 binding sites in promoter regions were averaged. The normalized enrichment ratios for overproduced non-degradable Cse4^K16R^ were normalized by the normalized enrichment ratios for overproduced normal Cse4. These normalized enrichments for non-degradable Cse4 were then plotted on a linear scale (means in arbitrary units (a.u.)+/−SEM). (B) ChIP-qPCR data depicting normalized enrichments for non-degradable Cse4 are presented for 6 previously-tested *CLR*s (Figure 2 and Figure S3) and for 6 non-*CLR* Cse4 binding sites at gene promoters determined by ChIP-Seq.
(TIF)

Figure S11 Localization of Cse4 to non-centromeric regions does not require the centromere chaperone Scm3. Cse4 binding at extra-centromeric sites is not greatly affected by the presence or absence of the essential chaperone Scm3 in Cse4 OP strains. (A–C) Cse4 ChIP-Seq binding profiles are compared in the presence (red) or absence (blue) of Scm3, upon overproduction of Cse4. Examples on chromosomes 1 (A), 6 (B) and 3 (C) are depicted. A 66% increase in the number of Cse4 non-centromeric binding sites was observed when *SCM3* was deleted. Despite this discrepancy, binding sites are highly correlated (Spearman's rho = 0.85; P<10^−15^). Control samples (immunoprecipitates from untagged strains) are shown in grey. Open reading frames (ORFs) are represented by purple boxes. Horizontal scale bars represent 1 kb. Significant regions of protein binding are represented by a like-colored box under the corresponding signal tracks. (D) Overlap of binding regions between Cse4 OP *SCM3* (red) and Cse4 OP *scm3Δ* (blue). Note that the Venn diagram is not drawn to scale.
(TIF)

Figure S12 Genomic features associated with *CLR*s are conserved in sequences homologous to *CLR*s in the *Saccharomyces sensu stricto*. Comparison of all sequences homologous to *CLR*s in *S. mikatae*, *S. kudriavzevii* and S. bayanus that were deemed conserved by blastn scores (Figure 4D) for association with *CEN*s (within 25 kb) (A), association with tRNAs (within 5 kb) (B), mean AT content of the most AT-rich 90-bp stretch of DNA (C), and average length of intergenic regions (D). Annotations and sequences were obtained from the *Saccharomyces sensu stricto* database [42]. Tests of significance followed the procedures taken for the comparison of *CLR*s and *CEN*s in *S. cerevisiae* (Figure 4A) and details are given in the Materials and Methods section (* p<0.05, ** p<0.01, *** p<0.001,. p<0.10).
(TIF)

Figure S13 Aggregated signal plots for individual kinetochore components at *CEN2*. Shown is ChIP-Seq signal for kinetochore proteins in Cse4 OP strains (blue) compared to WT (red). Plots depict the log ratio of read enrichment for Cse4 (A), Mif2 (B), Ndc10 (C) and Ndc80 (D), centered at *CEN2,* on log 2 scales.
(TIF)

Figure S14 Aggregated signal plots for individual kinetochore components at *CEN5*. Shown is ChIP-Seq signal for kinetochore proteins in Cse4 OP strains (blue) compared to WT (red). Plots depict the log ratio of read enrichment for Cse4 (A), Mif2 (B), Ndc10 (C) and Ndc80 (D), centered at *CEN5*, on log 2 scales.
(TIF)

Figure S15 Data used in this study fit multivariate normal distributions. Transformed data for 12 variables (Table S1) have been examined across *CLR*s and negative control regions (*LCNCR*s) using a χ^2^ distribution. (A–C) χ^2^ normal quantile plots, including data points (red), an ideal fit line (black) and 95% confidence intervals (blue), are presented for all 61 sites (*CLR*s and negative control regions) (A), for 23 *CLR*s (B), and for 38 negative control regions (C). On the horizontal axis are the theoretical quantiles, and on the vertical axis are the data quantiles.
(TIF)

Table S1 Raw data from 12 variables for 23 *CLR*s and 38 control regions (*LCNCR*s).
(DOC)

Table S2 Yeast strains used in this study.
(DOC)

Table S3 Primer sequences used for qPCR.
(DOC)
