# Supplementary figures and images for: Centromere-Like Regions in the Budding Yeast Genome
Source: PLoS Genet. 2013 Jan 17;9(1):e1003209. doi: 10.1371/journal.pgen.1003209 (PMC3547844; doi:10.1371/journal.pgen.1003209)

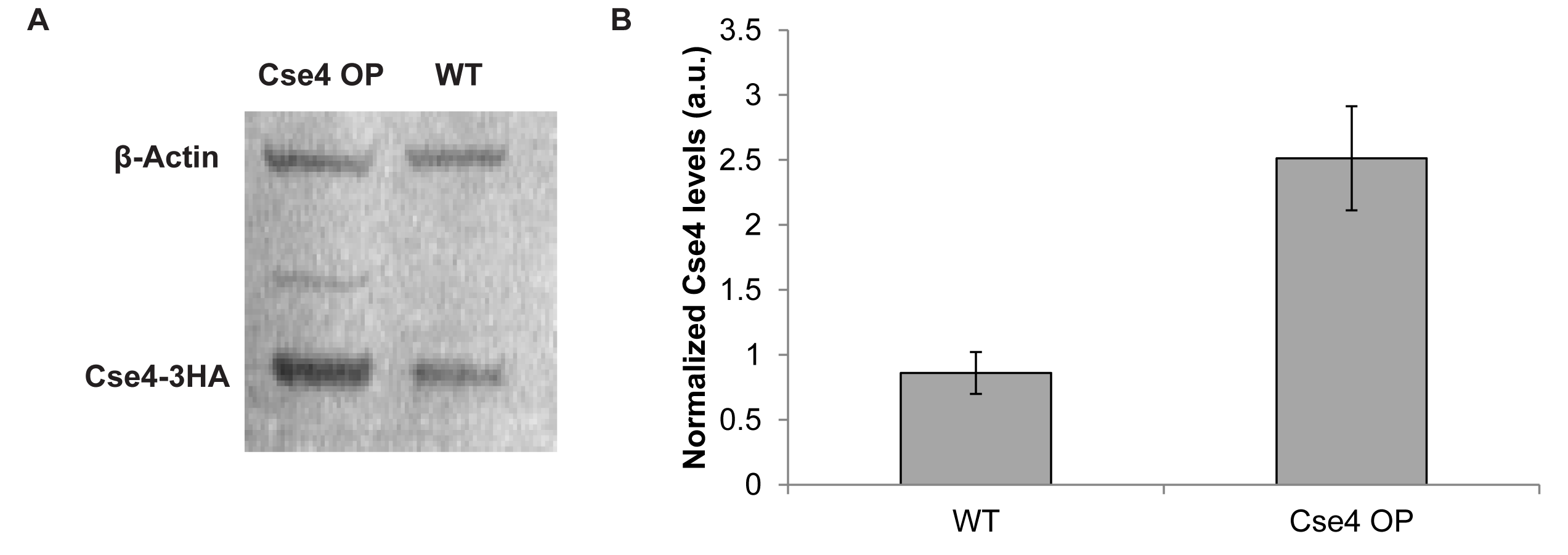

Supplement: Figure S1 — Western blot analysis of Cse4 levels in WT and Cse4 OP strains. (A) Western blot image showing the levels of Cse4-3HA (internal tag) in Cse4 OP and WT strains, as well as β-actin as a loading control. (B) Quantitation of Cse4 protein levels in WT and Cse4 OP strains. Cse4 abundance was normalized by the β-actin protein levels. Normalized Cse4 levels (means in arbitrary units (a.u.)+/−standard errors of the mean (SEM)) are plotted on a linear scale. Individual enrichments were obtained from four biological replicates. (TIF) [file pgen.1003209.s001.tif]

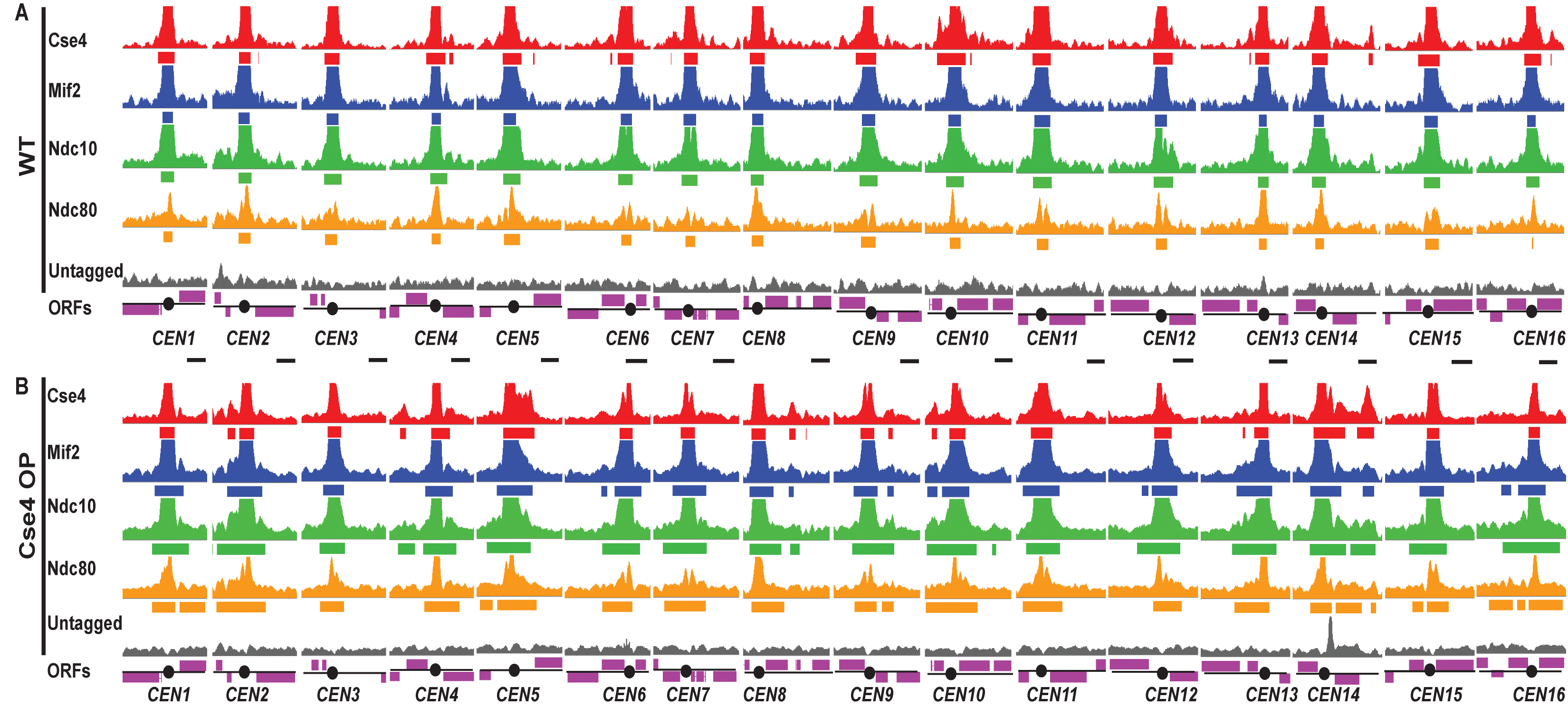

Supplement: Figure S2 — All CENs are occupied by four kinetochore proteins, in WT and Cse4 OP strains. ChIP-Seq signal tracks for Cse4 (red), Mif2 (blue), Ndc10 (green) and Ndc80 (orange) are scaled according to the number of uniquely-mapping reads, in WT (A) and Cse4 OP strains (B). Significant binding sites are represented by a liked-colored box under its corresponding signal track. Control samples (immunoprecipitates from untagged strains) are shown in grey. Open reading frames (ORFs) are represented by purple boxes. The black circles indicate centromeres. Horizontal scale bars represent 1 kb. (TIF) [file pgen.1003209.s002.tif]

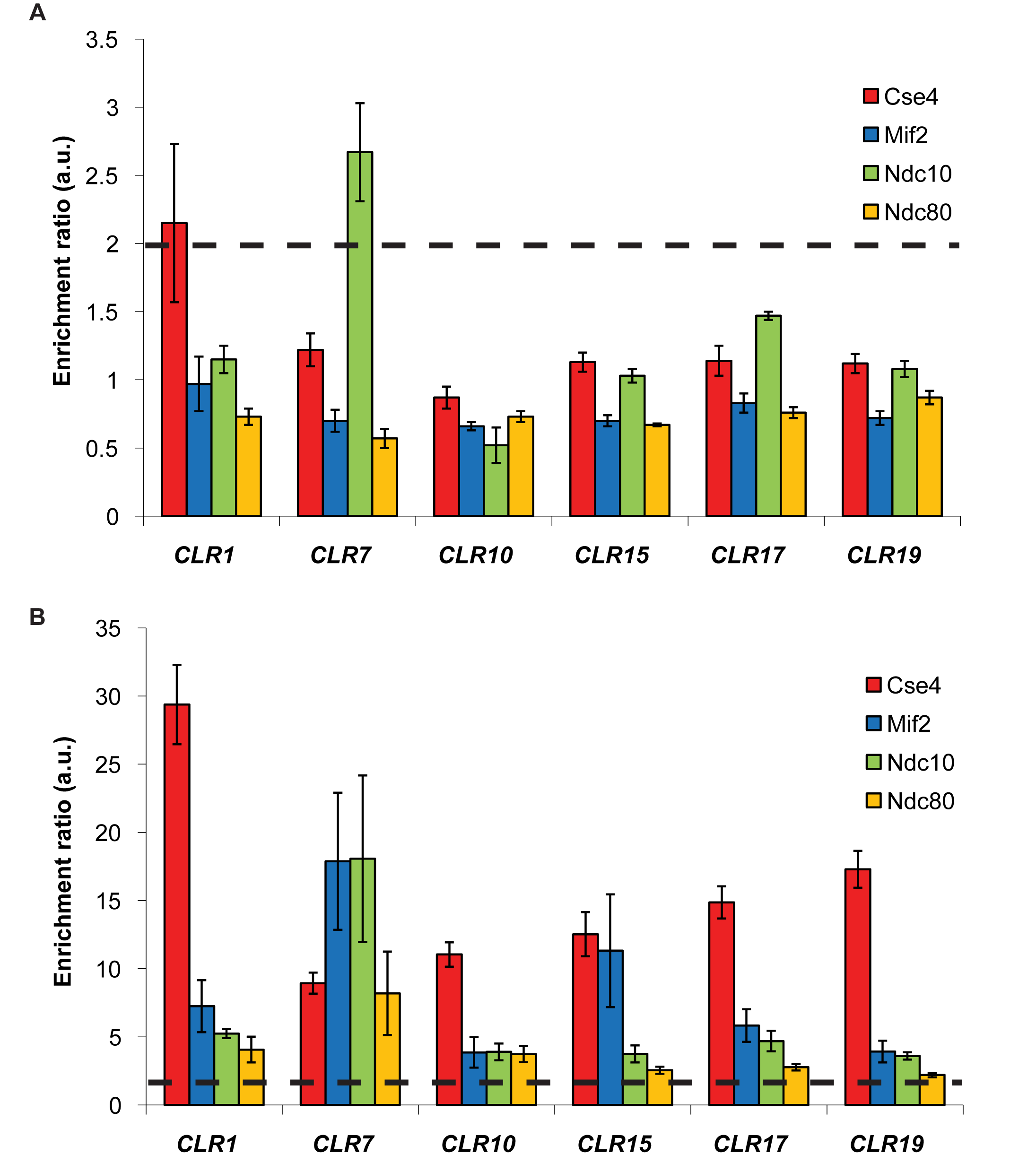

Supplement: Figure S3 — ChIP-qPCR validation of 6 CLRs. (A) All tested CLRs were not bound by all four proteins in WT. Only Cse4 in CLR1 and Ndc10 in CLR7 displayed significant binding (normalized enrichment ratio >2, dotted line). (B) In Cse4 OP, significantly enriched protein binding was confirmed at the six CLRs tested, for all four proteins examined (normalized enrichment ratio >2, dotted line). Normalized enrichment ratios (means in arbitrary units (a.u.)+/−standard errors of the mean (SEM)) are plotted on a linear scale. Individual enrichments were obtained from qPCR reactions run in triplicates and performed in at least two biological replicates. Note the different scales between (A) and (B). A normalized enrichment of 1 indicates no enrichment over a negative control region not enriched for kinetochore proteins. (TIF) [file pgen.1003209.s003.tif]

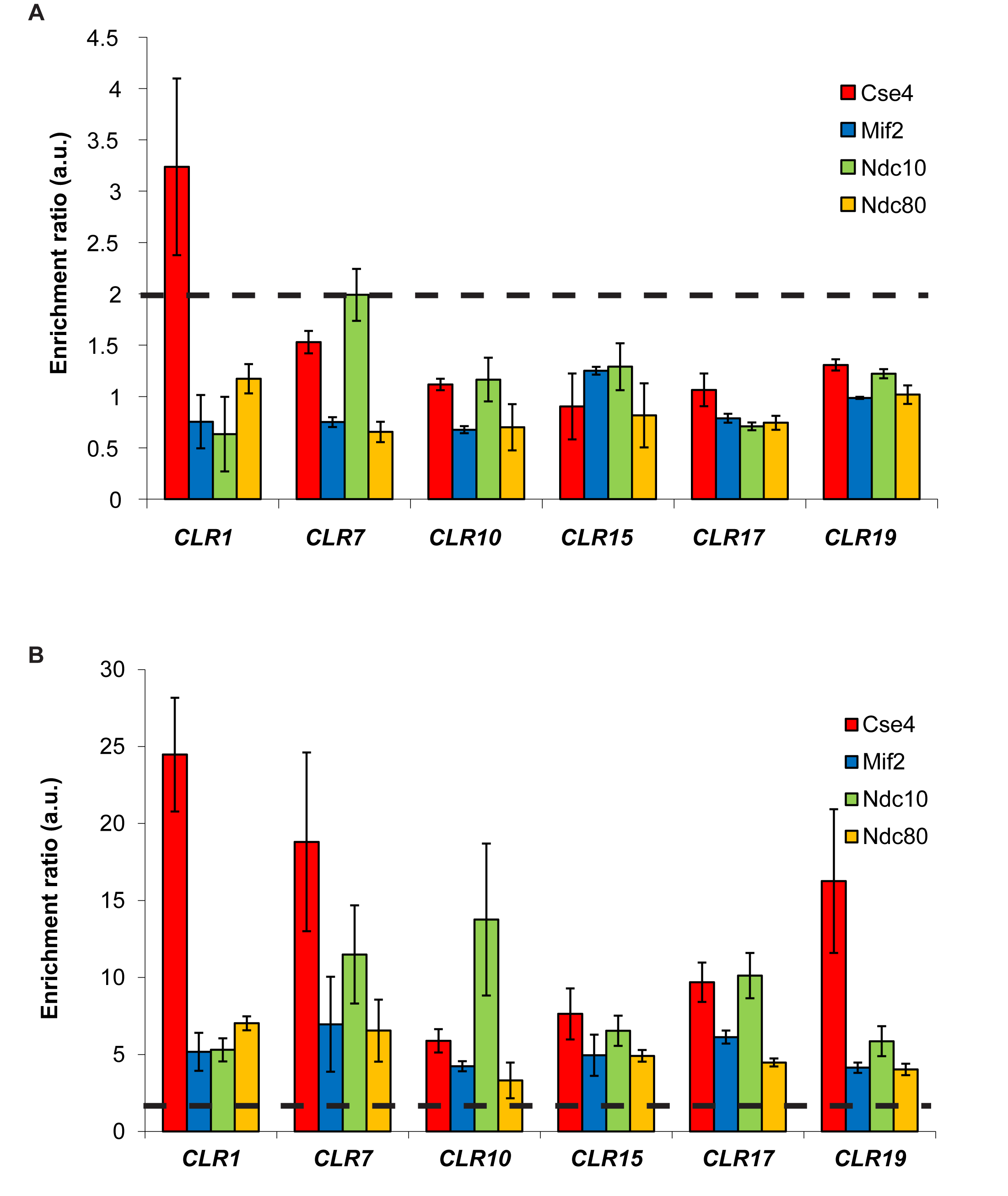

Supplement: Figure S4 — Kinetochore proteins are present at CLRs when the pericentric intramolecular C loop is abrogated in a cohesin-deficient mcd1-1 background. (A) All tested CLRs were not bound by all four proteins in strains with normal Cse4 levels (WT) in a mcd1-1 background, similarly to Figure S3A. Only Cse4 in CLR1 and Ndc10 in CLR7 displayed significant binding (normalized enrichment ratio >2, dotted line). (B) In strains with elevated Cse4 levels (Cse4 OP) in a mcd1-1 background, significantly enriched protein binding was confirmed at the six CLRs tested, for all four proteins examined (normalized enrichment ratio >2, dotted line), similarly to Figure S3B. Normalized enrichment ratios (means in arbitrary units (a.u.)+/−standard errors of the mean (SEM)) are plotted on a linear scale. Individual enrichments were obtained from qPCR reactions run in triplicates and performed in at least two biological replicates. Note the different scales between (A) and (B). A normalized enrichment of 1 indicates no enrichment over a negative control region not enriched for kinetochore proteins. (TIF) [file pgen.1003209.s004.tif]

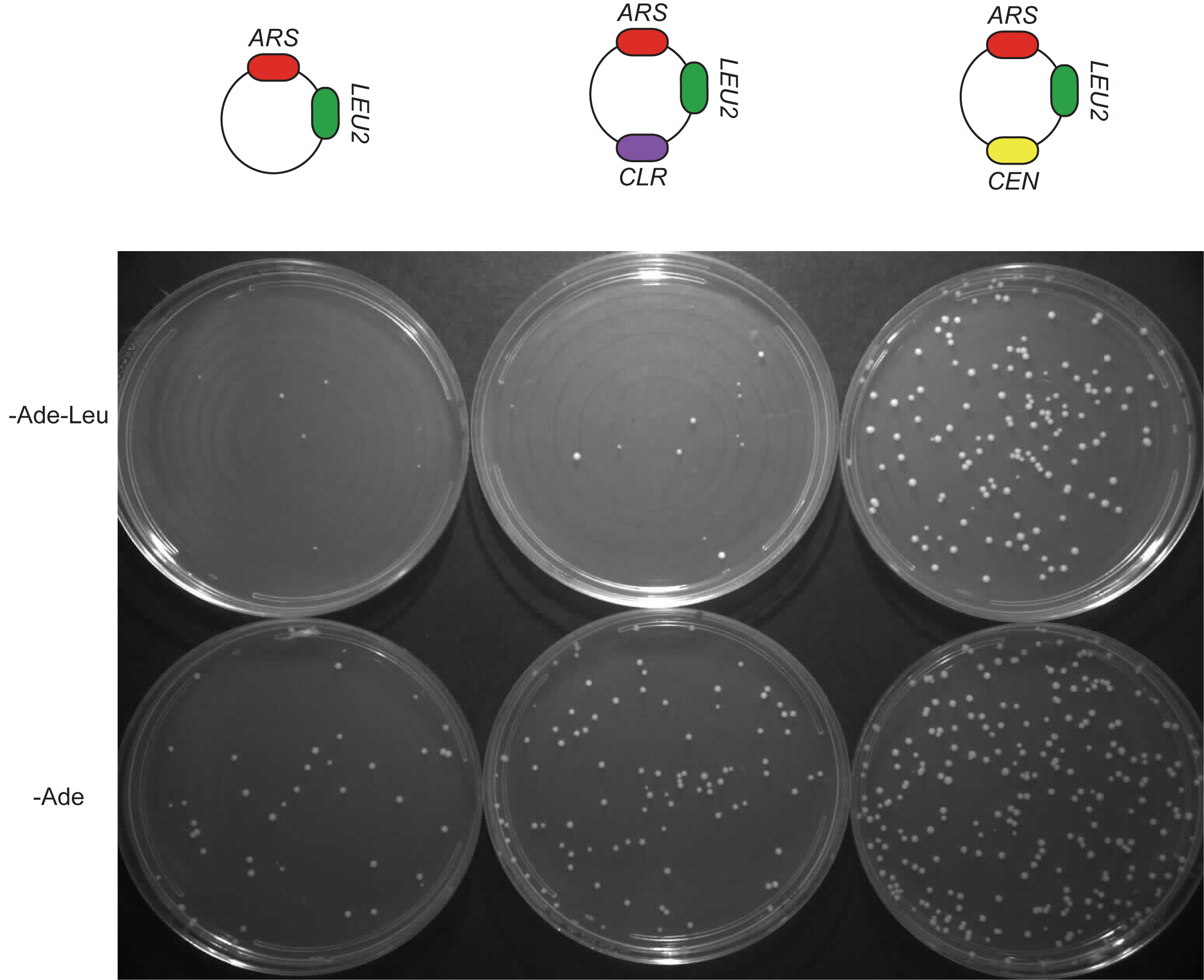

Supplement: Figure S5 — Colony formation on plates from plating assays. Strains bearing CLR plasmids generate colonies of intermediate size, which are on average larger than those carrying ARS plasmids and smaller than those containing CEN plasmids. After a few generations of growth in non-selective rich medium, strains carrying various plasmids were plated on selective medium (-Ade -Leu) for four days. Strains plated on permissive medium (-Ade) do not differ in colony size. (TIF) [file pgen.1003209.s005.tif]

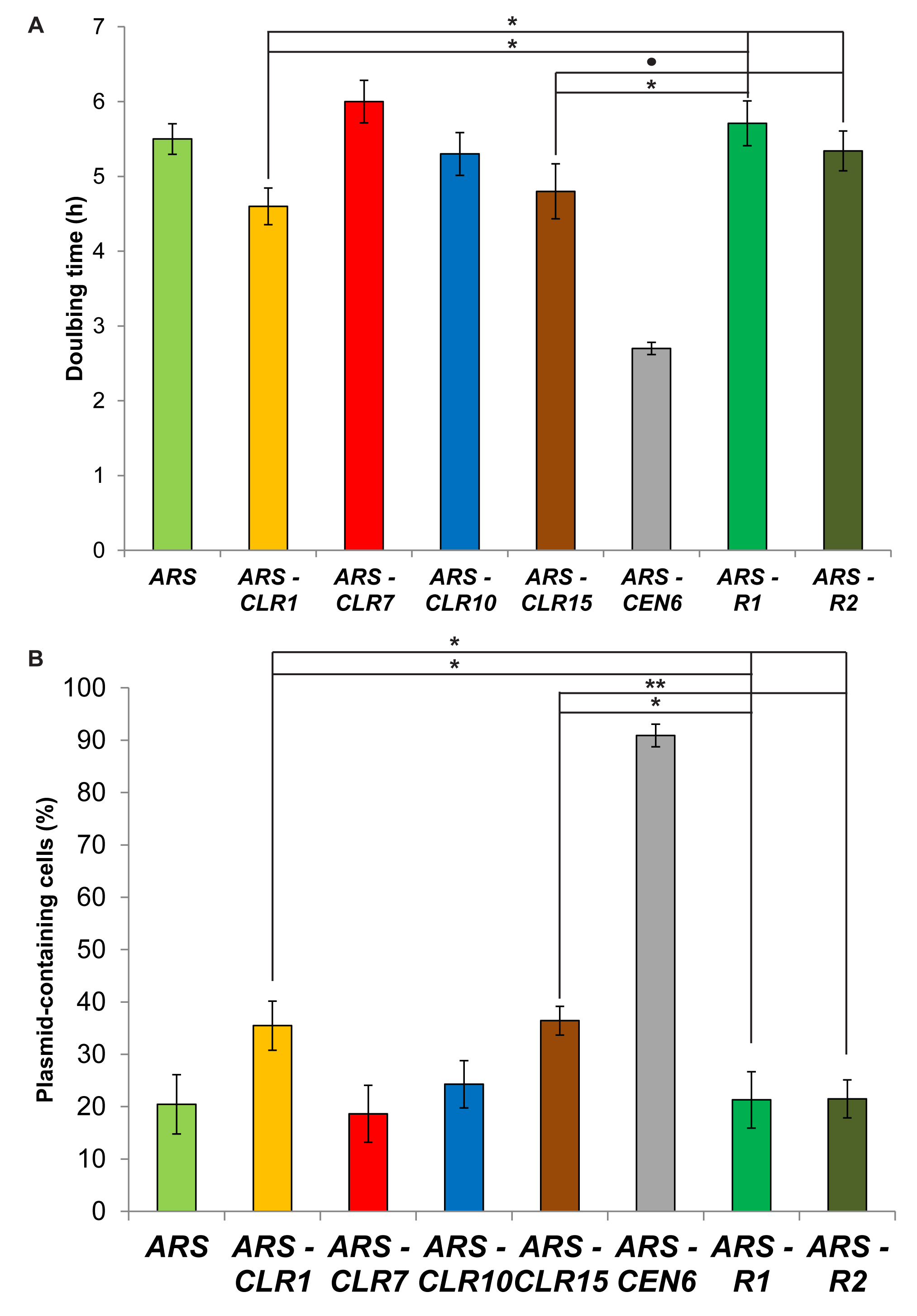

Supplement: Figure S6 — Comparison of ARS-CLR plasmids and ARS plasmids bearing random inserts of similar sizes in plasmid segregation assays. (A) Doubling times in selective medium of strains carrying different plasmids (Means+/−SEM). (B) Fraction of plasmid-containing cells after growth in non-selective media for strains bearing various plasmids (Means+/−SEM). P-values were computed using MCMC simulations (* p<0.05, ** p<0.01, *** p<0.001,. p<0.10) (A–B). ARS-R1 and ARS-R2 refer to ARS plasmids bearing random inserts of 1 kb and 0.8 kb, respectively. (TIF) [file pgen.1003209.s006.tif]

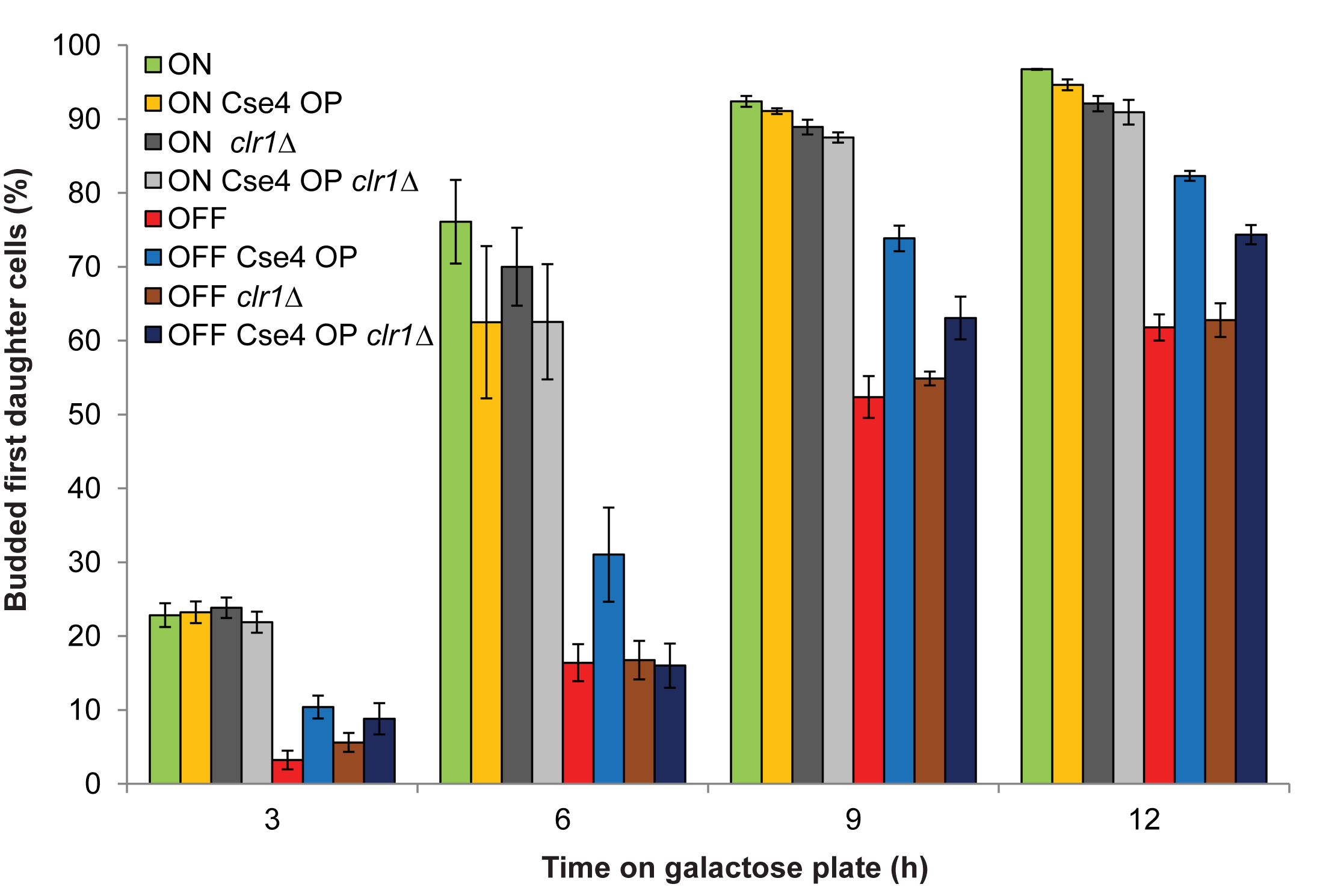

Supplement: Figure S7 — Time course analysis of daughter cell budding after transfer of mother cells to a galactose plate. Values (Means+/−SEM) are given for various genotypes at 3-h intervals (refer to Figure 3D). ON and OFF indicate the presence of the conditional CEN3. (TIF) [file pgen.1003209.s007.tif]

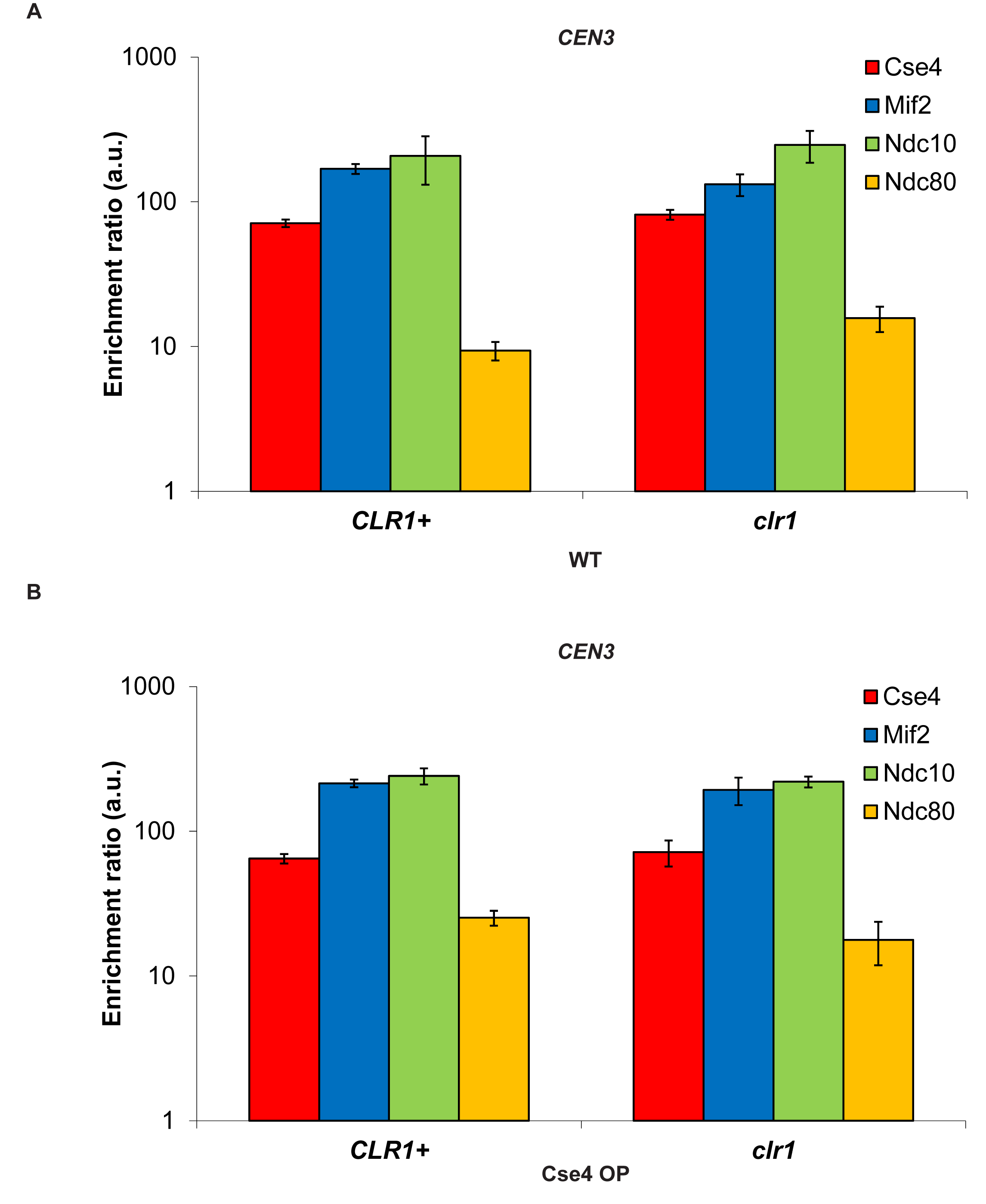

Supplement: Figure S8 — Binding levels of kinetochore proteins at CEN3 are similar in clr1 and CLR1+ strains. ChIP-qPCR confirms that the presence of kinetochore proteins at CEN3 is not affected when CLR1 is deleted. Individual protein enrichments at CEN3 were normalized and compared in strains containing CLR1 (CLR1+) and missing CLR1 (clr1), for WT (A) and Cse4 OP (B). Normalized enrichment ratios (means in arbitrary units (a.u.)+/−SEM) were plotted on a log 10 scale. A normalized enrichment of 1 indicates no enrichment over a negative control region not enriched for kinetochore proteins. (TIF) [file pgen.1003209.s008.tif]

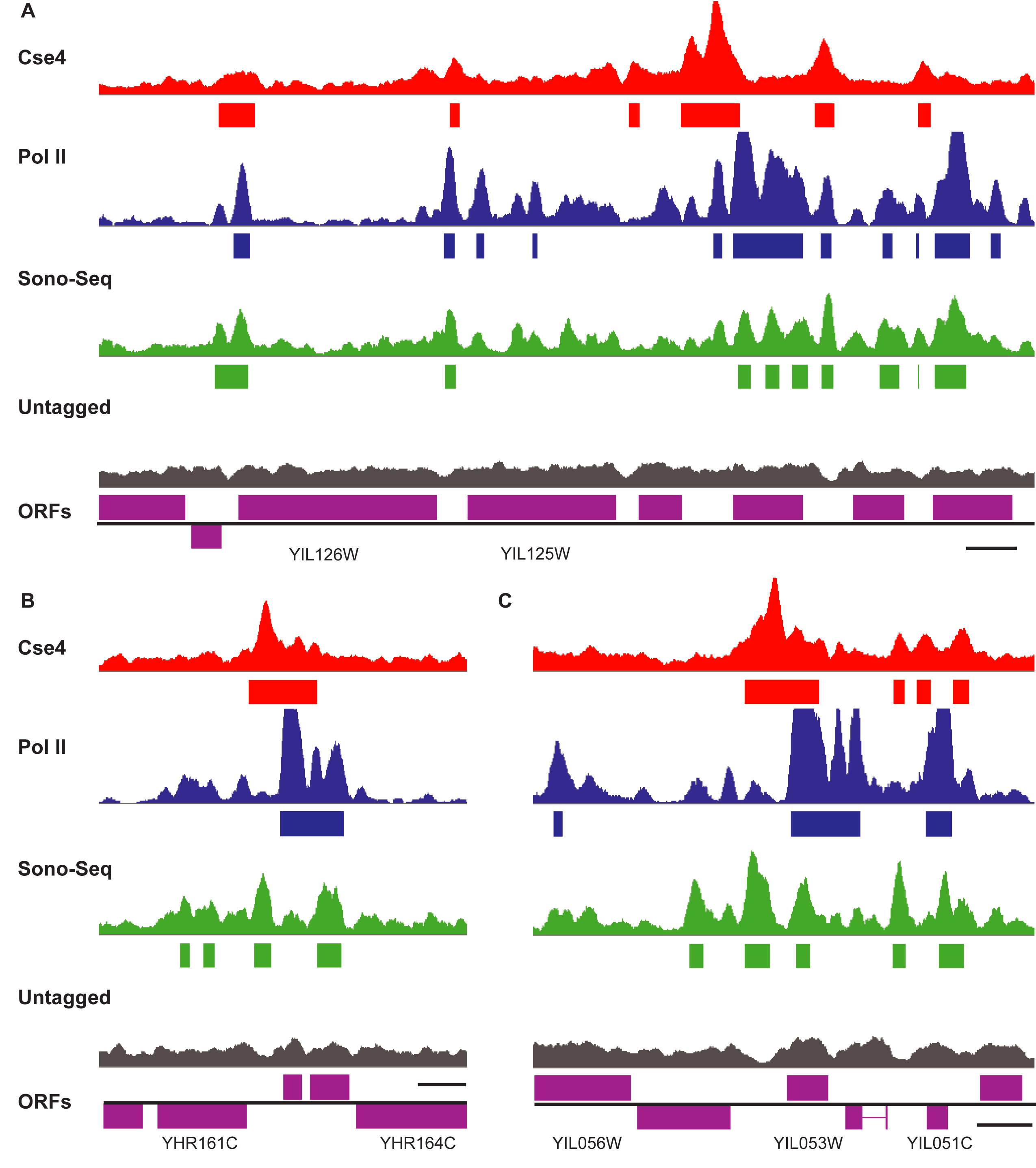

Supplement: Figure S9 — Cse4 marks a subset of open chromatin. Cse4 is associated with promoters, accessible chromatin and RNA polymerase II-bound regions. Cse4 shows a broader euchromatin distribution upon its overproduction, consistent with previous reports [62]. (A) On chromosome 9, regions of Cse4 (red) binding overlap promoters, regions bound by RNA polymerase II (blue) and Sono-Seq (green) sites. Sono-Seq regions are enriched for open chromatin [34], [51]. Promoter nucleosomes and regions of high histone turnover have been associated with higher levels of non-centromeric Cse4 [37], [63]. Cse4 binding is also correlated with overlapping or adjacent RNA polymerase II occupancy (Spearman's rho = 0.32; P<10−8), in concordance with the presence of Cse4 around transcribed regions [5], [12]. (B–C) An extra-centromeric Cse4 binding region most commonly overlaps open chromatin (i.e. promoters and Sono-Seq region), and is adjacent to an ORF bound by RNA polymerase II. Examples on chromosomes 8 (B) and 9 (C) are shown. Control samples (immunoprecipitates from untagged strains) are shown in grey. Open reading frames (ORFs) are represented by purple boxes. Horizontal scale bars represent 1 kb. Significant regions of protein binding or sensitivity to Sono-Seq are represented by a like-colored box under the corresponding signal tracks. (TIF) [file pgen.1003209.s009.tif]

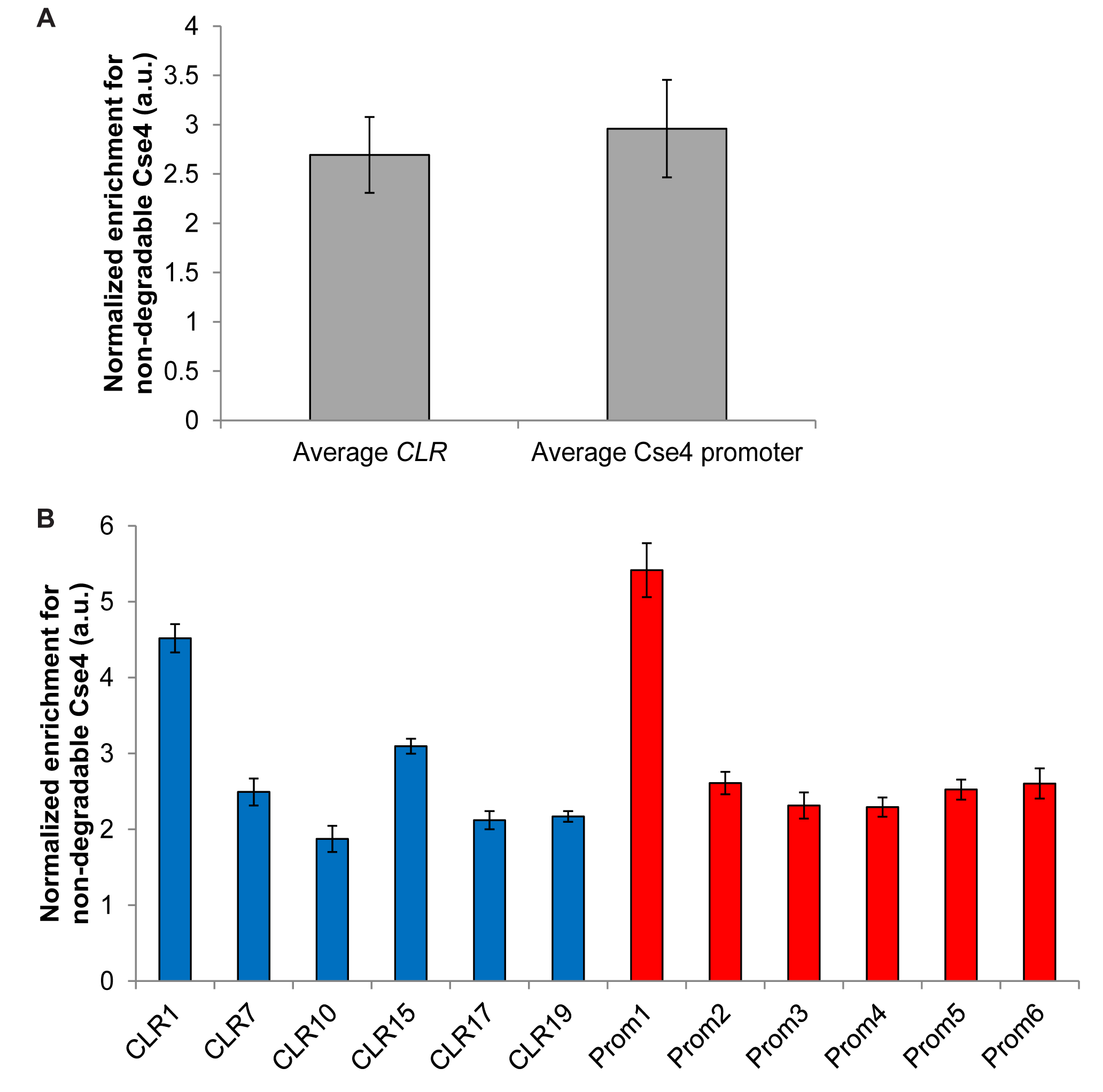

Supplement: Figure S10 — Non-degradable Cse4 is not preferentially enriched at CLRs compared to non-CLR Cse4 binding sites at gene promoters. (A) ChIP-qPCR comparative analyses of CLRs and non-CLR promoters bound by Cse4 indicate that non-degradable Cse4K16R is relatively more abundant than normal Cse4 at both CLRs and non-CLR Cse4 binding sites, in similar proportions. Non-degradable Cse4 enrichments for 6 CLRs (same as Figure 2 and Figure S3) and for 6 non-CLR Cse4 binding sites in promoter regions were averaged. The normalized enrichment ratios for overproduced non-degradable Cse4K16R were normalized by the normalized enrichment ratios for overproduced normal Cse4. These normalized enrichments for non-degradable Cse4 were then plotted on a linear scale (means in arbitrary units (a.u.)+/−SEM). (B) ChIP-qPCR data depicting normalized enrichments for non-degradable Cse4 are presented for 6 previously-tested CLRs (Figure 2 and Figure S3) and for 6 non-CLR Cse4 binding sites at gene promoters determined by ChIP-Seq. (TIF) [file pgen.1003209.s010.tif]

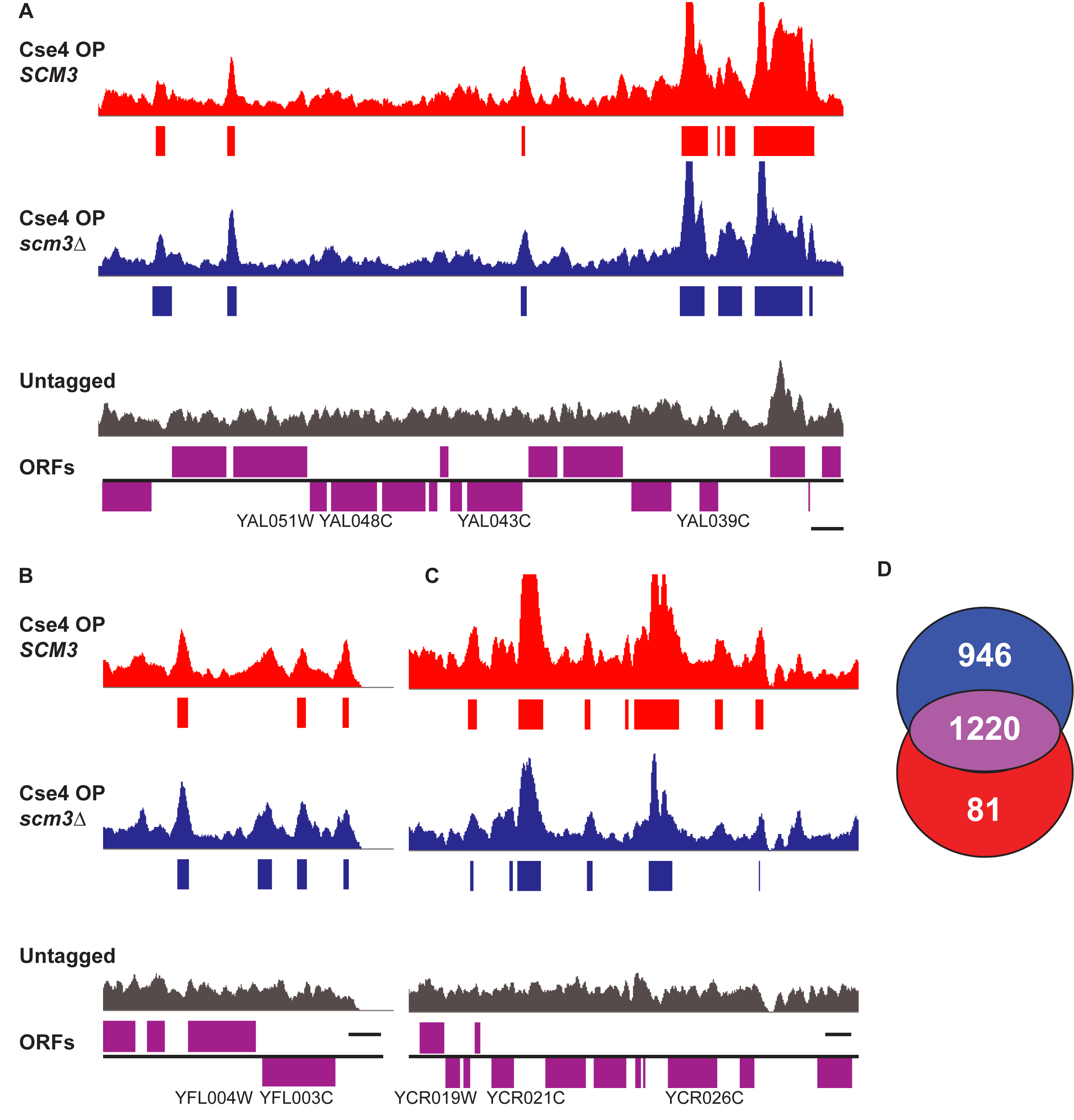

Supplement: Figure S11 — Localization of Cse4 to non-centromeric regions does not require the centromere chaperone Scm3. Cse4 binding at extra-centromeric sites is not greatly affected by the presence or absence of the essential chaperone Scm3 in Cse4 OP strains. (A–C) Cse4 ChIP-Seq binding profiles are compared in the presence (red) or absence (blue) of Scm3, upon overproduction of Cse4. Examples on chromosomes 1 (A), 6 (B) and 3 (C) are depicted. A 66% increase in the number of Cse4 non-centromeric binding sites was observed when SCM3 was deleted. Despite this discrepancy, binding sites are highly correlated (Spearman's rho = 0.85; P<10−15). Control samples (immunoprecipitates from untagged strains) are shown in grey. Open reading frames (ORFs) are represented by purple boxes. Horizontal scale bars represent 1 kb. Significant regions of protein binding are represented by a like-colored box under the corresponding signal tracks. (D) Overlap of binding regions between Cse4 OP SCM3 (red) and Cse4 OP scm3Δ (blue). Note that the Venn diagram is not drawn to scale. (TIF) [file pgen.1003209.s011.tif]

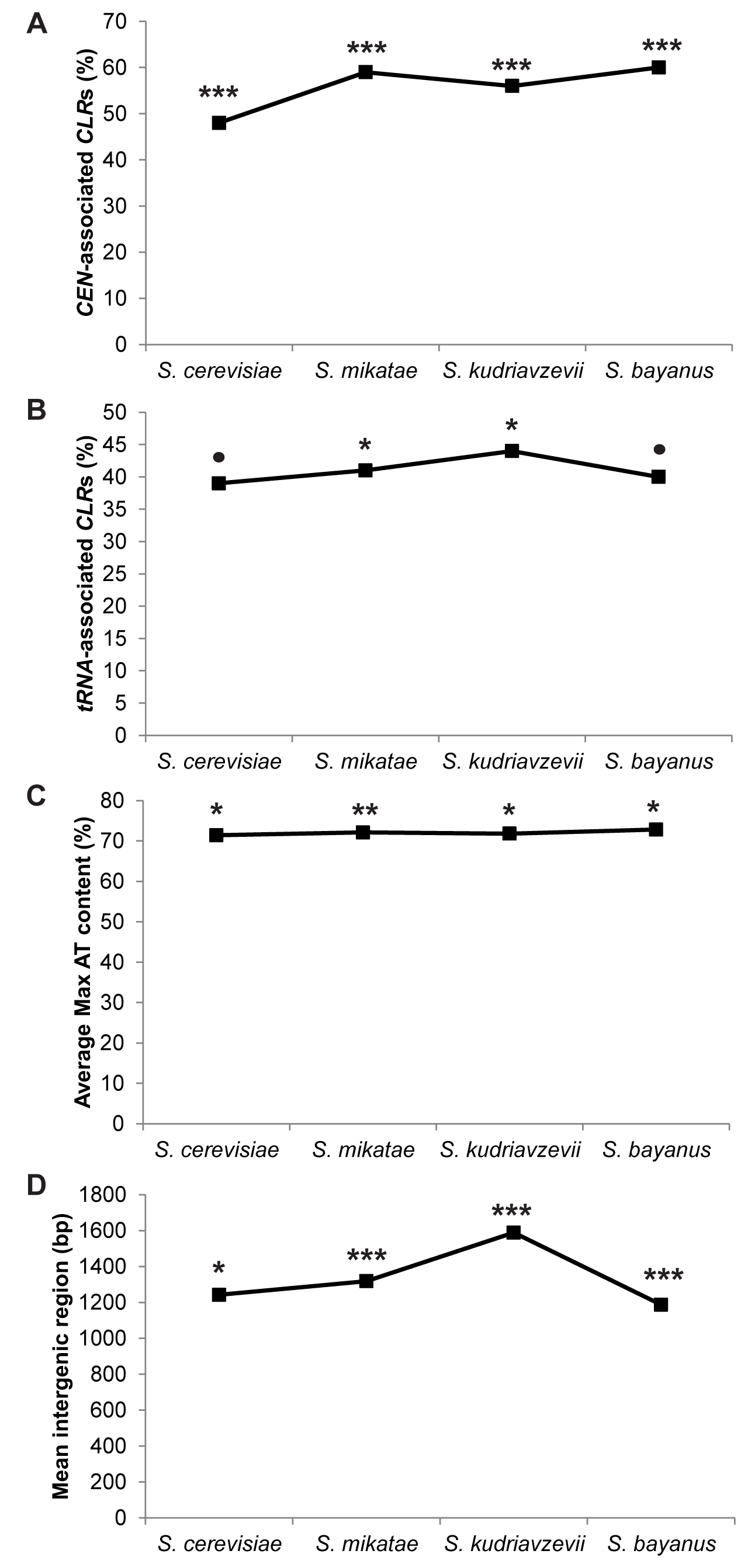

Supplement: Figure S12 — Genomic features associated with CLRs are conserved in sequences homologous to CLRs in the Saccharomyces sensu stricto. Comparison of all sequences homologous to CLRs in S. mikatae, S. kudriavzevii and S. bayanus that were deemed conserved by blastn scores (Figure 4D) for association with CENs (within 25 kb) (A), association with tRNAs (within 5 kb) (B), mean AT content of the most AT-rich 90-bp stretch of DNA (C), and average length of intergenic regions (D). Annotations and sequences were obtained from the Saccharomyces sensu stricto database [42]. Tests of significance followed the procedures taken for the comparison of CLRs and CENs in S. cerevisiae (Figure 4A) and details are given in the Materials and Methods section (* p<0.05, ** p<0.01, *** p<0.001,. p<0.10). (TIF) [file pgen.1003209.s012.tif]

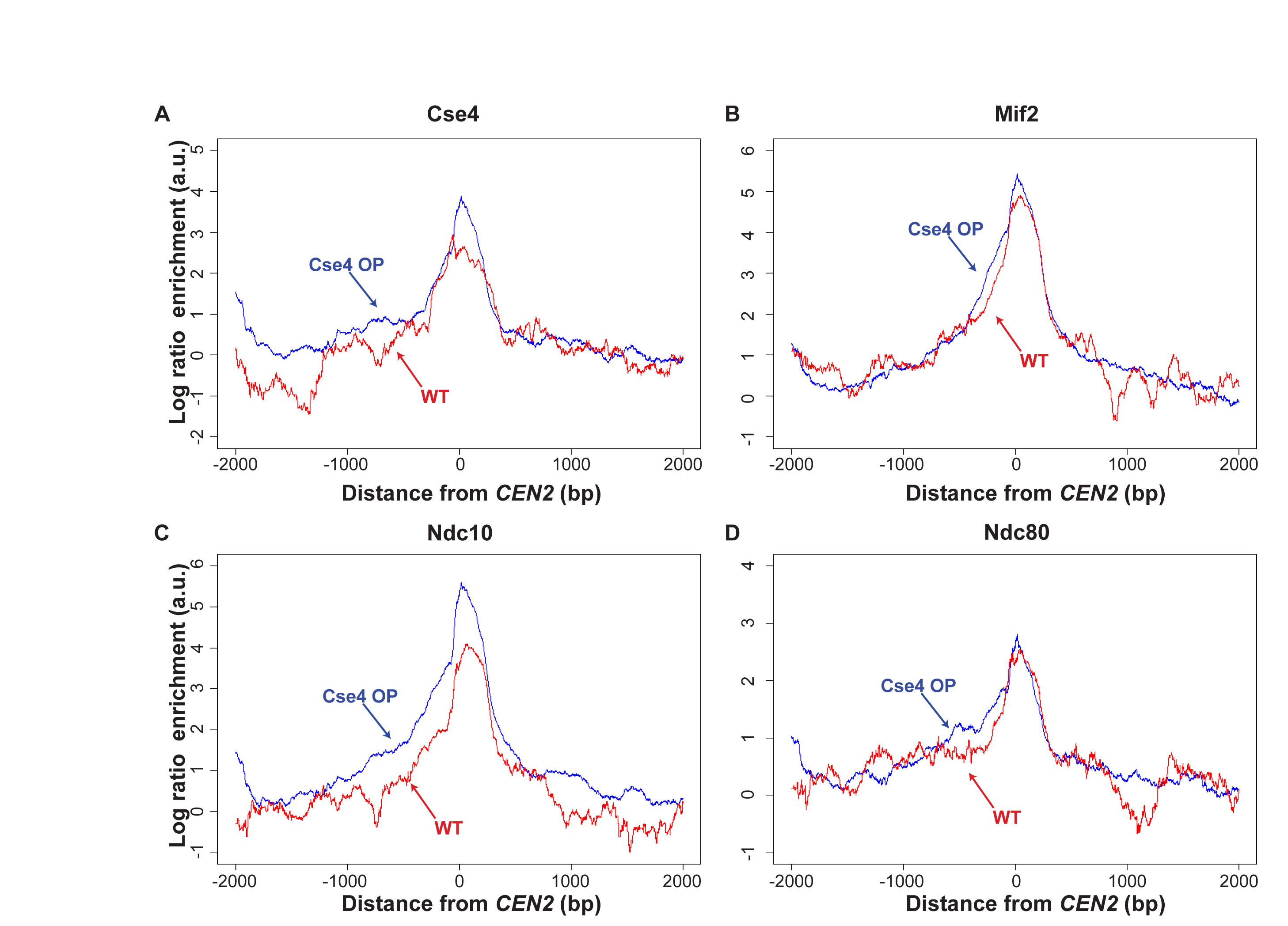

Supplement: Figure S13 — Aggregated signal plots for individual kinetochore components at CEN2. Shown is ChIP-Seq signal for kinetochore proteins in Cse4 OP strains (blue) compared to WT (red). Plots depict the log ratio of read enrichment for Cse4 (A), Mif2 (B), Ndc10 (C) and Ndc80 (D), centered at CEN2, on log 2 scales. (TIF) [file pgen.1003209.s013.tif]

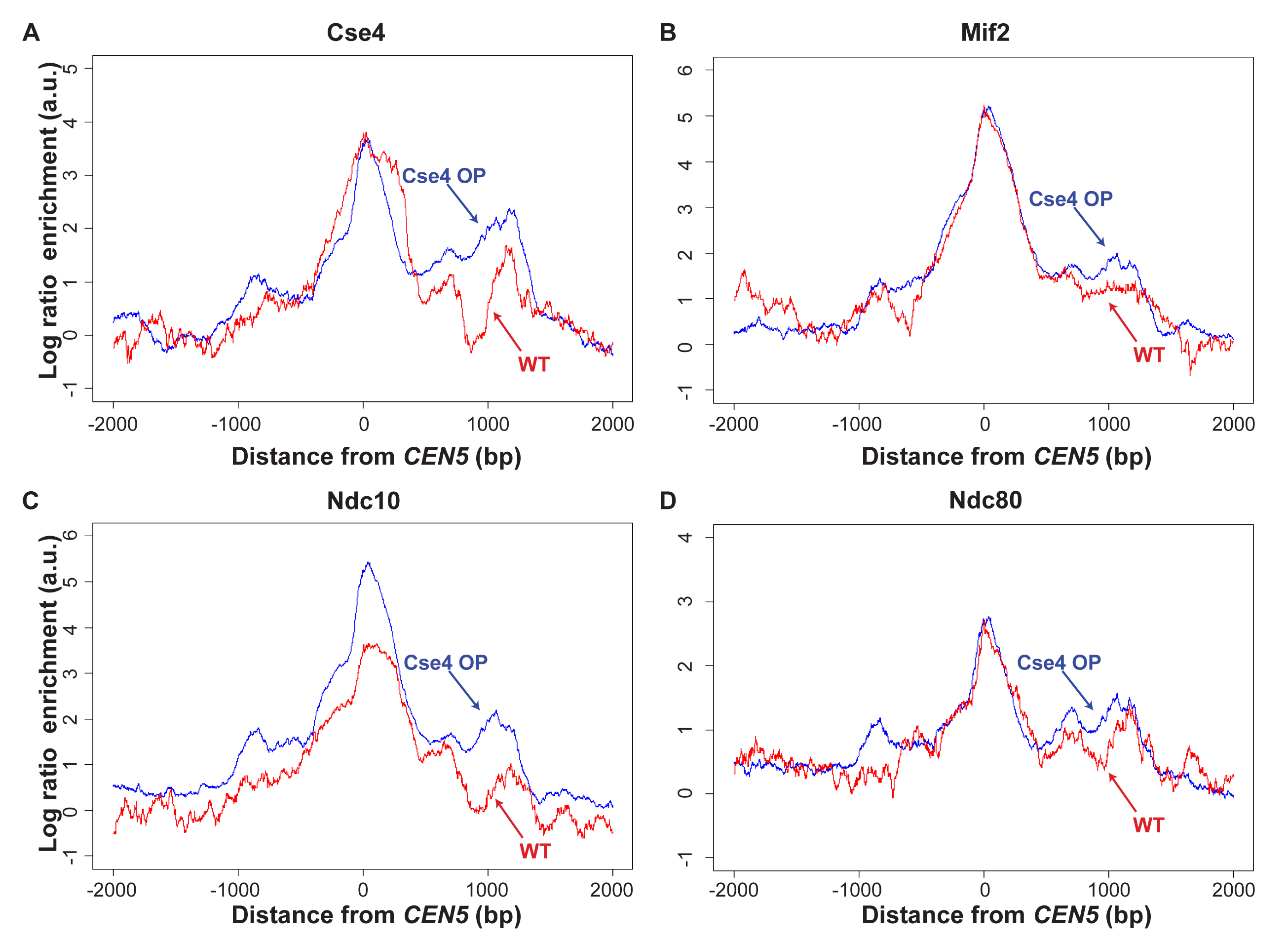

Supplement: Figure S14 — Aggregated signal plots for individual kinetochore components at CEN5. Shown is ChIP-Seq signal for kinetochore proteins in Cse4 OP strains (blue) compared to WT (red). Plots depict the log ratio of read enrichment for Cse4 (A), Mif2 (B), Ndc10 (C) and Ndc80 (D), centered at CEN5, on log 2 scales. (TIF) [file pgen.1003209.s014.tif]

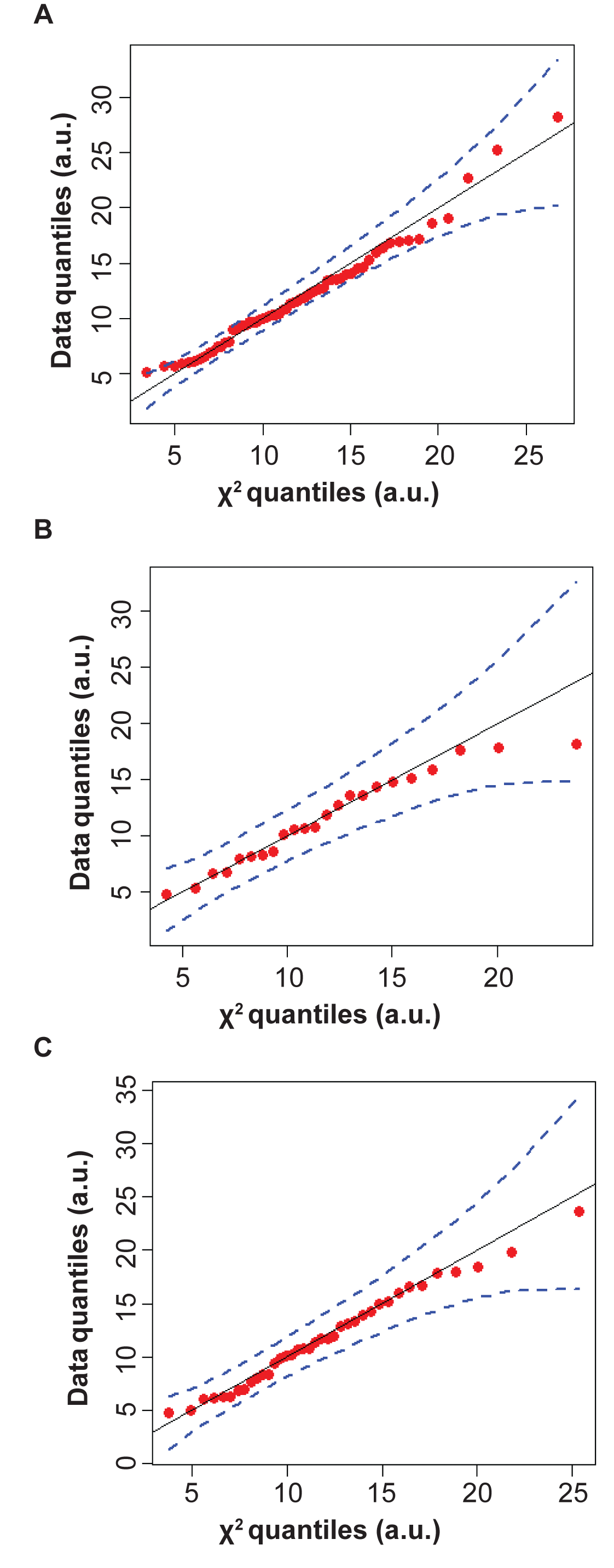

Supplement: Figure S15 — Data used in this study fit multivariate normal distributions. Transformed data for 12 variables (Table S1) have been examined across CLRs and negative control regions (LCNCRs) using a χ2 distribution. (A–C) χ2 normal quantile plots, including data points (red), an ideal fit line (black) and 95% confidence intervals (blue), are presented for all 61 sites (CLRs and negative control regions) (A), for 23 CLRs (B), and for 38 negative control regions (C). On the horizontal axis are the theoretical quantiles, and on the vertical axis are the data quantiles. (TIF) [file pgen.1003209.s015.tif]
