# Supplementary material for: Centromere-Like Regions in the Budding Yeast Genome
Source: PLoS Genet. 2013 Jan 17;9(1):e1003209. doi: 10.1371/journal.pgen.1003209 (PMC3547844; doi:10.1371/journal.pgen.1003209)
Supplement: Table S1 — Raw data from 12 variables for 23 CLRs and 38 control regions (LCNCRs). (DOC) [file pgen.1003209.s016.doc]

**Table S1. Raw data from 12 variables for 23 *CLR*s and 38 control regions (*LCNCR***s).

| **Site** | **V1 (bp)** | **V2 (kb)** | **V3 (kb)** | **V4 (kb)** | **V5 (%)** | **V6 (%)** | **V7 (bp)** | **V8 (rank)** | **V9 (rank)** | **V10 (rank)** | **V11 (%)** | **V12 (rank)** |
| --- | --- | --- | --- | --- | --- | --- | --- | --- | --- | --- | --- | --- |
| ***CLR1*** | 8857 | 8 | 0.2 | 0.2 | 60.5 | 73.3 | 3008 | 1572 | 1697 | 2298 | 0.00 | 2745 |
| ***CLR2*** | 2783 | 2.8 | 0.6 | 0.2 | 62.5 | 75.6 | 1527 | 3199 | 2413 | 3448 | 0.00 | 731 |
| ***CLR3*** | 72931 | 4.8 | 3.8 | 14.5 | 52.8 | 68.9 | 672 | 365.5 | 434.5 | 2400 | 15.71 | 1458 |
| ***CLR4*** | 38210 | 19.3 | 14 | 16.3 | 52.7 | 64.4 | 1896 | 70 | 83 | 1302 | 0.00 | 265 |
| ***CLR5*** | 1213 | 0.1 | 12.4 | 20.9 | 65.1 | 78.9 | 520 | 744 | 4430 | 2819 | 26.43 | 1398 |
| ***CLR6*** | 3610 | 8.7 | 12.7 | 17.8 | 61.1 | 74.4 | 616 | 354 | 278 | 250 | 1.40 | 95 |
| ***CLR7*** | 563934 | 2.3 | 2.6 | 8.7 | 51.6 | 70 | 758 | 644 | 1232 | 218 | 0.00 | 1724 |
| ***CLR8*** | 1145 | 11.5 | 11.5 | 11.5 | 65.1 | 73.3 | 1202 | 3135 | 5289.5 | 1958.5 | 48.83 | 847 |
| ***CLR9*** | 11046 | 4.5 | 2.4 | 2.4 | 59.5 | 74.4 | 308 | 373 | 317 | 527 | 63.33 | 369 |
| ***CLR10*** | 9524 | 4.7 | 2.5 | 2.5 | 63.1 | 78.9 | 743 | 2664 | 5324 | 1415.5 | 0.00 | 2124 |
| ***CLR11*** | 20155 | 6.6 | 6.6 | 6.6 | 55.2 | 68.9 | 841 | 2444 | 3719 | 2223 | 0.00 | 1092 |
| ***CLR12*** | 208988 | 17.6 | 10.6 | 11.6 | 60.9 | 75.6 | 2347 | 358 | 263 | 2808 | 0.00 | 2228 |
| ***CLR13*** | 15234 | 1.8 | 22.2 | 18 | 53.2 | 63.3 | 536 | 572 | 736 | 309 | 41.50 | 853 |
| ***CLR14*** | 651657 | 1.5 | 0.5 | 0.5 | 55.3 | 70 | 3759 | 98 | 125 | 1272 | 0.00 | 409 |
| ***CLR15*** | 78112 | 1.3 | 0.3 | 1.3 | 52.5 | 70 | 889 | 64 | 44 | 85 | 100.00 | 236 |
| ***CLR16*** | 238735 | 8.4 | 71 | 127 | 60 | 78.9 | 1234 | 712 | 946 | 1377 | 53.83 | 2550 |
| ***CLR17*** | 14290 | 9.2 | 5.2 | 36.2 | 60.9 | 74.4 | 576 | 472 | 665 | 968 | 15.71 | 576 |
| ***CLR18*** | 266834 | 8 | 85.2 | 160.2 | 53.6 | 64.4 | 817 | 698 | 1485 | 2515 | 14.38 | 634 |
| ***CLR19*** | 12979 | 0.9 | 43.2 | 51 | 59.6 | 73.3 | 1786 | 400 | 324 | 3 | 47.13 | 40 |
| ***CLR20*** | 483210 | 6.2 | 3.3 | 16.2 | 62.2 | 73.3 | 721 | 689 | 1223 | 483 | 0.00 | 2793 |
| ***CLR21*** | 153754 | 9.6 | 5.6 | 5.6 | 57.8 | 71.1 | 731 | 765 | 792 | 1714 | 12.83 | 28 |
| ***CLR22*** | 143469 | 0.2 | 95.9 | 94.9 | 47.9 | 61.1 | 1220 | 466 | 548 | 2253 | 6.80 | 4249 |
| ***CLR23*** | 225812 | 18 | 87 | 1 | 55.1 | 65.6 | 4559 | 762 | 434 | 3082 | 0.00 | 3425 |
| ***LCNCR1*** | 503921 | 0.3 | 2.3 | 68.9 | 53.3 | 60 | 1315 | 267 | 597 | 788.5 | 96.63 | 1142 |
| ***LCNCR2*** | 104931 | 0.7 | 27.5 | 46.5 | 57.3 | 60 | 1622 | 951 | 4579 | 49 | 86.06 | 3495 |
| ***LCNCR3*** | 23157 | 5.6 | 2.9 | 3.9 | 53.2 | 60 | 1305 | 778 | 1308 | 51 | 86.87 | 34 |
| ***LCNCR4*** | 166243 | 6.6 | 45.4 | 36.4 | 64.6 | 85.6 | 1048 | 2430 | 1748 | 26 | 89.70 | 36 |
| ***LCNCR5*** | 112888 | 1.7 | 13 | 12 | 55.1 | 62.2 | 1081 | 1390 | 1800 | 14 | 77.23 | 30 |
| ***LCNCR6*** | 386321 | 4 | 5.3 | 7.3 | 54.1 | 60 | 1000 | 1761 | 3280 | 1 | 72.86 | 3587.5 |
| ***LCNCR7*** | 7345 | 16.7 | 13.3 | 6.3 | 60.6 | 75.6 | 454 | 141 | 301 | 1385 | 84.40 | 83 |
| ***LCNCR8*** | 4115 | 4.1 | 8.1 | 8.1 | 58.7 | 71.1 | 1027 | 1033.5 | 711 | 803 | 68.33 | 717 |
| ***LCNCR9*** | 22043 | 16.6 | 0.3 | 1.3 | 55.7 | 65.6 | 1096 | 2127 | 3650 | 2198.5 | 97.82 | 1245 |
| ***LCNCR10*** | 10921 | 0.2 | 22.4 | 26.4 | 62.1 | 73.3 | 256 | 201 | 4283 | 1834.5 | 42.00 | 3010.5 |
| ***LCNCR11*** | 89585 | 11.6 | 23.6 | 73.6 | 64.8 | 90 | 205 | 103 | 465 | 327 | 28.33 | 151.5 |
| ***LCNCR12*** | 275588 | 10.5 | 1.5 | 0.5 | 54.3 | 61.1 | 787 | 2171 | 2191.5 | 30 | 71.36 | 227 |
| ***LCNCR13*** | 207910 | 0.4 | 2.4 | 3.4 | 55 | 63.3 | 1711 | 519 | 914 | 797 | 100.00 | 1646 |
| ***LCNCR14*** | 139854 | 4 | 3.9 | 3.9 | 65.2 | 74.4 | 862 | 2631 | 2802.5 | 1542 | 60.27 | 2583 |
| ***LCNCR15*** | 308812 | 0.7 | 11.7 | 10.7 | 58.7 | 68.9 | 151 | 5 | 18 | 196 | 0.00 | 39 |
| ***LCNCR16*** | 81112 | 1 | 18 | 11 | 62.2 | 73.3 | 1693 | 1961 | 3459.5 | 25 | 68.16 | 2473 |
| ***LCNCR17*** | 105934 | 0.7 | 11.4 | 34.7 | 66 | 82.2 | 748 | 2352 | 2370 | 53 | 82.89 | 207 |
| ***LCNCR18*** | 453257 | 1.8 | 69.9 | 69.9 | 60.4 | 74.4 | 316 | 720 | 1057 | 21 | 44.46 | 3218 |
| ***LCNCR19*** | 135566 | 2.8 | 44.8 | 119.8 | 60.3 | 72.2 | 1799 | 1864.5 | 2357 | 335 | 83.27 | 2426 |
| ***LCNCR20*** | 219612 | 2 | 3 | 3 | 47.8 | 58.9 | 334 | 61 | 75 | 335.5 | 86.40 | 2823 |
| ***LCNCR21*** | 35835 | 19.1 | 81 | 8.1 | 61.8 | 72.2 | 1102 | 1898 | 4994 | 6 | 68.36 | 2859.7 |
| ***LCNCR22*** | 46890 | 36 | 24 | 18 | 56 | 75.6 | 592 | 106 | 139 | 69 | 49.17 | 46 |
| ***LCNCR23*** | 593010 | 24.1 | 23.1 | 24.1 | 65.7 | 76.7 | 1639 | 844 | 1810 | 346 | 0.00 | 1642 |
| ***LCNCR24*** | 345655 | 4.3 | 14.3 | 11.3 | 52.4 | 57.8 | 1315 | 1274.5 | 997 | 66 | 93.79 | 2624 |
| ***LCNCR25*** | 139212 | 1.1 | 76.1 | 69.1 | 56.9 | 67.8 | 1726 | 925 | 3301 | 11 | 80.92 | 3752 |
| ***LCNCR26*** | 93688 | 16.4 | 32.5 | 41.4 | 60.7 | 67.8 | 691 | 2421 | 2190 | 1818 | 58.64 | 2561 |
| ***LCNCR27*** | 78224 | 1.7 | 65.7 | 49.7 | 54.3 | 61.1 | 1516 | 2257 | 1660 | 93 | 93.88 | 31 |
| ***LCNCR28*** | 135335 | 3.2 | 22.6 | 23.6 | 55.4 | 64.4 | 1831 | 652 | 5142 | 41 | 83.14 | 976.5 |
| ***LCNCR29*** | 374235 | 8 | 19 | 29 | 54.9 | 61.1 | 1666 | 1799 | 1163 | 239 | 93.33 | 221 |
| ***LCNCR30*** | 218712 | 2.6 | 3.6 | 3.6 | 57.3 | 71.1 | 403 | 61 | 75 | 105 | 51.25 | 242 |
| ***LCNCR31*** | 68917 | 1.1 | 0.2 | 1.1 | 57.6 | 70 | 667 | 588 | 645 | 1083 | 12.29 | 1314.5 |
| ***LCNCR32*** | 150934 | 26.8 | 17.8 | 42.8 | 52 | 58.9 | 1018 | 564 | 817 | 10 | 67.87 | 206 |
| ***LCNCR33*** | 913812 | 40.7 | 12.7 | 12.7 | 48.2 | 60 | 2640 | 1019 | 1133 | 1913 | 0.00 | 3953 |
| ***LCNCR34*** | 292743 | 2.1 | 77.1 | 11.4 | 59.8 | 68.9 | 2905 | 1509 | 4028 | 45 | 49.02 | 27 |
| ***LCNCR35*** | 13146 | 2.1 | 0.4 | 0.4 | 64.9 | 81.1 | 847 | 373 | 3394 | 59 | 0.00 | 446 |
| ***LCNCR36*** | 11834 | 0.4 | 23.6 | 24.6 | 58.4 | 68.9 | 762 | 1004.5 | 1330.5 | 481 | 75.80 | 761 |
| ***LCNCR37*** | 12146 | 1.1 | 1 | 1 | 59.3 | 66.7 | 847 | 373 | 3394 | 59 | 84.78 | 446 |
| ***LCNCR38*** | 686612 | 43.5 | 19.5 | 19.5 | 53.3 | 61.1 | 1189 | 22 | 3592 | 63 | 65.94 | 26 |

V1-V4=Distance from *CEN* (V1), *ARS* (V2), tRNA (V3), and retrotransposon (V4, LTR), V5=mean AT content, V6=max AT content of 90-bp stretch of DNA, V7=Length of closest intergenic (interORF) region, V8-V9=Rank of closest Sono-Seq region, scored against HA (V8) or against Myc (V9), V10=Rank of closest PolII binding site, V11=Percent of length of binding site overlapping ORF, V12=Rank of closest Cse4 binding site in Cse4 WT strain.
