# Supplementary material for: Centromere-Like Regions in the Budding Yeast Genome
Source: PLoS Genet. 2013 Jan 17;9(1):e1003209. doi: 10.1371/journal.pgen.1003209 (PMC3547844; doi:10.1371/journal.pgen.1003209)
Supplement: Table S2 — Yeast strains used in this study. (DOC) [file pgen.1003209.s017.doc]

**Table S2.** Yeast strains used in this study.

| **Strain** | **Genotype** | **Parent strain** | **Source** |
| --- | --- | --- | --- |
| PL76-6C | Same as CMY675-3C but CSE4-3HAinternal-KanMX4 | W303 | This study |
| PL78-1B | Same as CMY675-3C but TRP1-P_GAL1_-CSE4 | W303 | This study |
| PL79-1C | Same as CMY675-3C but TRP1-P_GAL1_-CSE4-3HAinternal-KanMX4 | W303 | This study; Meluh et al., 1998 |
| PL82-7B | Same as CMY675-3C but TRP1-P_GAL1_-CSE4 scm3::HphMX4 | W303 | This study |
| PL85-5B | Same as CMY675-3C but TRP1-P_GAL1_-CSE4-3HAi-KanMX4 scm3::HphMX4 | W303 | This study |
| CMY675-3C | MAT a ADE2 his3-11,15 leu2-3,112 lys2 met17 trp1-1 ura3-1 can1-100 | W303 | Christopher Yellman, unpublished |
| CMY1841-6C | Same as CMY675-3C but NDC10-9XMyc-KanMX4 | W303 | Christopher Yellman, unpublished |
| PL86-1D | Same as CMY675-3C but TRP1-P_GAL1_-CSE4 NDC10-9XMyc-KanMX4 | W303 | This study |
| PL90-6C | Same as CMY675-3C but MAT α MIF2-9XMyc-KanMX4 | W303 | This study |
| PL91-1D | Same as CMY675-3C but NDC80-9XMyc-KanMX4 | W303 | This study |
| PL97-4A | Same as CMY675-3C but MAT α TRP1-P_GAL1_-CSE4 MIF2-9XMyc-KanMX4 | W303 | This study |
| PL98-2D | Same as CMY675-3C but TRP1-P_GAL1_-CSE4 NDC80-9XMyc-KanMX4 | W303 | This study |
| PL107-2D | Same as CMY675-3C but ChrXIV_632000_:TRP1-P_GAL1_-CSE4 | W303 | This study |
| PL108-3A | Same as CMY675-3C but P_GAL1_-CEN3-URA3*_K. lactis_* | W303 | Reid et al., 2008 |
| PL108-10A | Same as PL107-2D but P_GAL1_-CEN3-URA3*_K. lactis_* | W303 | This study |
| SLY806 | MAT α P_HIS3_-GFP-LacI2-HIS3 LEU2:LacO256-LEU2 P_GAL1_-CEN3-TRP1 ChrIII_116000_:URA3 ade2-1 his3-11,15 leu2-3,112 lys2 met17 | W303 | Lacefield et al., 2009 |
| PL120-13C | MAT a P_HIS3_-GFP-LacI2-HIS3 LEU2:LacO256-LEU2 ade2-1 his3-11,15 leu2-3,112 lys2 met17 ADE2 ChrXIV_632000_:TRP1-P_GAL1_-CSE4 | W303 | This study |
| PL122-3D | MAT a P_HIS3_-GFP-LacI2-HIS3 LEU2:LacO256-LEU2 P_GAL1_-CEN3-TRP1 ChrIII_116000_:URA3 ade2-1 his3-11,15 leu2-3,112 lys2 met17 P_HIS3_-GFP-LacI_2_-ADE2 cdc15-2 | W303 | Lacefield et al., 2009 |
| PL122-11C | Same as PL122-3D but ChrXIV_632000_:TRP1-P_GAL1_-CSE4 | W303 | This study |
| PL126-1 | Same as PL108-3A but ChrIII_123000-124000_::NatMX4 | W303 | This study |
| PL127-1 | Same as PL108-3A but ChrIII_123000-124000_::HphMX4 | W303 | This study |
| PL128-2 | Same as PL108-10A but ChrIII_123000-124000_::NatMX4 | W303 | This study |
| PL129-2 | Same as PL108-10A but ChrIII_123000-124000_::HphMX4 | W303 | This study |
| PL130-1 | Same as PL122-3D but ChrIII_123000-124000_::NatMX4 | W303 | This study |
| PL131-5 | Same as PL122-11D but ChrIII_123000-124000_::HphMX4 | W303 | This study |
| PL125-315 | Same as PL107-2D + pRS315 (LEU2, CEN6, ARS) | W303 | This study; Sikorski et al., 1989 |
| PL125-26 | Same as PL107-2D + pPL26 (LEU2, ARS1) | W303 | This study |
| PL125-33 | Same as PL107-2D + pPL33 (LEU2, ARS1, ChrI_141500-142000_) | W303 | This study |
| PL125-34 | Same as PL107-2D + pPL34 (LEU2, ARS1, ChrIII_123300-124000_) | W303 | This study |
| PL125-39 | Same as PL107-2D + pPL39 (LEU2, ARS1, ChrXI_518300-518900_) | W303 | This study |
| PL125-41 | Same as PL107-2D + pPL41 (LEU2, ARS1, ChrIV_1013700-1014300_) | W303 | This study |
| PL125-43 | Same as PL107-2D + pPL43 (LEU2, ARS1, CEN6) | W303 | This study |
| PL125-53 | Same as PL107-2D + pPL53 (LEU2, ARS1, ChrII_130200-131200_) | W303 | This study |
| PL125-56 | Same as PL107-2D + pPL56 (LEU2, ARS1, ChrX_630300-631100_) | W303 | This study |
| PL145-1 | Same as PL76-6C but ChrIII_123000-124000_::HphMX4 | W303 | This study |
| PL146-2 | Same as CMY1841-6C but ChrIII_123000-124000_::HphMX4 | W303 | This study |
| PL147-1 | Same as PL90-6C but ChrIII_123000-124000_::HphMX4 | W303 | This study |
| PL148-2 | Same as PL91-1D but ChrIII_123000-124000_::HphMX4 | W303 | This study |
| PL149-1 | Same as PL97-4A but ChrIII_123000-124000_::HphMX4 | W303 | This study |
| PL150-1 | Same as PL86-1D but ChrIII_123000-124000_::HphMX4 | W303 | This study |
| PL151-7 | Same as PL79-1C but ChrIII_123000-124000_::HphMX4 | W303 | This study |
| PL152-3 | Same as PL98-2D but ChrIII_123000-124000_::HphMX4 | W303 | This study |
| PL153-17C | Same as PL76-6C but MAT α *mcd1-1* | W303 | This study |
| PL154-1A | Same as PL79-1C but MAT α *mcd1-1* | W303 | This study |
| PL155-10B | Same as PL78-1B but *mcd1-1* | W303 | This study |
| PL155-11B | Same as PL86-1D but MAT α *mcd1-1* | W303 | This study |
| PL156-9B | Same as PL90-6C but MAT a *mcd1-1* | W303 | This study |
| PL157-14A | Same as PL91-1D but *mcd1-1* | W303 | This study |
| PL158-10D | Same as PL97-4A but MAT a *mcd1-1* | W303 | This study |
| PL159-19A | Same as PL78-1B but *mcd1-1* | W303 | This study |
| PL159-19C | Same as PL98-2D but *mcd1-1* | W303 | This study |
| PL160-19B | Same as CMY1841-6C but MAT α *mcd1-1* | W303 | This study |
| PL160-19C | Same as CMY675-3C but *mcd1-1* | W303 | This study |
| SBY1071 | Same as CMY675-3C but *ura3-1*: P_GAL1_- CSE4-13XMyc-URA3 and *ade2-1* | W303 | Collins et al., 2004 |
| SBY1813 | Same as PL78-1B but CSE4:CSE4-12XMyc-URA3 and *ade2-1* | W303 | Collins et al., 2004 |
| SBY3570 | Same as CMY675-3C + pSB816(2 micron, P_GAL1_- CSE4-13XMyc, URA3) but *ade2-1* | W303 | Collins et al., 2004 |
| SBY3571 | Same as CMY675-3C + pSB816(2 micron, P_GAL1_- CSE4-13XMyc, URA3) but *ade2-1* | W303 | Collins et al., 2004 |
| PL161-40C | Same as PL122-3D but *ndc10-1* | W303 | This study |
| PL162-14C | Same as PL122-11C but *ndc10-1* | W303 | This study |
| PL162-37D | Same as PL122-3D but *ndc10-1* | W303 | This study |
| PL162-52A | Same as PL122-11C but *ndc10-1* | W303 | This study |
