# Supplementary material for: Centromere-Like Regions in the Budding Yeast Genome
Source: PLoS Genet. 2013 Jan 17;9(1):e1003209. doi: 10.1371/journal.pgen.1003209 (PMC3547844; doi:10.1371/journal.pgen.1003209)
Supplement: Table S3 — Primer sequences used for qPCR. (DOC) [file pgen.1003209.s018.doc]

**Table S3. Primer sequences used for qPCR.**

| **Pair name** | **Primer name** | **Sequence** |
| --- | --- | --- |
| CLR1Q | CLR1Qfor | GCCATCGTCATTGACCTTTT |
| CLR1Q | CLR1Qrev | TTCGTGTCTGTTTCGCAGTC |
| CLR7Q | CLR7Qfor | ATAACCAGGACCCCCTTTTG |
| CLR7Q | CLR7Qrev | CAAAACCGATCTCGGAACAT |
| CLR10Q | CLR10Qfor | CCTTGAAGTCCTCGAAACGA |
| CLR10Q | CLR10Qrev | TCATCATTGTCAACGGCATT |
| CLR15Q | CLR15Qfor | ACACAGCGTGCCTAGTTCCT |
| CLR15Q | CLR15Qrev | CCCGAATCAGGAGAAACAAA |
| CLR17Q | CLR17Qfor | AAAACGCATGCTTCGTAAGG |
| CLR17Q | CLR17Qrev | CAAAATTTAGGGCCGAACAA |
| CLR19Q | CLR19Qfor | GAGACCGGGTTTGTTTTCAA |
| CLR19Q | CLR19Qrev | TCGGAACGGAAGCTAGAAGA |
| Cen3p1 | Cen3p1for | GATCAGCGCCAAACAATATGGAAAATCC |
| Cen3p1 | Cen3p1rev | AACTTCCACCAGTAAACGTTTCATATATCC |
| Neg1p1 | Neg1p1for | TCGCAATCTCAATGATTTGG |
| Neg1p1 | Neg1p1rev | CCTCCAAGCCCAGAGTTGTA |
| Neg1p2 | Neg1p2for | CCGGTGCTAAACAACCTGAT |
| Neg1p2 | Neg1p2rev | TTTAGTCAACGGTGGTGCTG |
| Prom1 | Prom1for | AAGGGGGAAAGAACCAAAGA |
| Prom1 | Prom1rev | ACTACGGCACAGAAGCCAAG |
| Prom2 | Prom2for | AGAGACGCGTTCAAGGAAGA |
| Prom2 | Prom2rev | CACGCTGCCTTTACTCCATT |
| Prom3 | Prom3for | GGCTCAGCGAAGATGACATT |
| Prom3 | Prom3rev | TCAGTGCCAAATTGGTGTCT |
| Prom4 | Prom4for | AGAGAGGTGGGGGAATTGAG |
| Prom4 | Prom4rev | TACCACCAAGCATCAACACC |
| Prom5 | Prom5for | GTGCAACAGGAAAACGAGGT |
| Prom5 | Prom5rev | AGTGCGGATTCATTTCCAAG |
| Prom6 | Prom6for | TTAACAACAACGGCAGCAAC |
| Prom6 | Prom6rev | TAAGGAAGCAGAGCCAAAGC |
